# Haul-Out Behaviour of the World's Northernmost Population of Harbour Seals (*Phoca vitulina*) throughout the Year

**DOI:** 10.1371/journal.pone.0086055

**Published:** 2014-01-22

**Authors:** Charmain D. Hamilton, Christian Lydersen, Rolf A. Ims, Kit M. Kovacs

**Affiliations:** 1 Norwegian Polar Institute, Fram Centre, Tromsø, Norway; 2 Department of Arctic and Marine Biology, University of Tromsø, Tromsø, Norway; Università degli Studi di Napoli Federico II, Italy

## Abstract

The harbour seal population in Svalbard occurs at the northernmost limit of the species' range. It experiences environmental extremes far beyond the norm for this species, including an extended period of polar night and extensive sea ice cover. In 2009 and 2010, 60 harbour seals (30 pups + 30 immature/mature seals) from this population were equipped with Satellite-Relay Data Loggers (SRDLs) to study their haul-out behaviour, with a special focus on the winter period. Using a combination of Generalized Additive Mixed Models and Cox Proportional Hazard models, the influences of sex, maturity, temporal, spatial and environmental factors on haul-out behaviour were explored. All of the seals continued to haul out even through the coldest periods during the polar night, though clear seasonality in the time spent hauled out daily was displayed by both immature and mature seals. Time spent hauled out daily decreased from ∼5.2 hrs in September to ∼1.2 hrs in February in these age groups, while pups displayed less seasonality (∼2.4 hrs/day throughout most of the year). The average at-sea period also exhibited seasonality, increasing to a maximum of ∼1.6 days in February (monthly maxima for individual animals ranged from 7 **to** 19 days). The seals showed a strong preference to haul out at low tide when hauling out on land but not when using sea ice as a haul-out platform. A diel rhythm in haul-out behaviour was present during the months with day–night cycling and midnight sun but not during the polar night. Haul-out behaviour was impacted to a greater extent by air pressure, through its effect on wind speed, than by absolute temperature values. The extreme environment in Svalbard likely causes some physiological challenges that might impact survival rates negatively, particularly among pups. Climate warming is likely to have positive effects on Svalbard's harbour seal population.

## Introduction

A strong dichotomy exists in the life history of all pinniped (true seals, fur seals, sea lions and walrus) species, with marine foraging dominating many aspects of their lives, though they remain tied to solid substrates (land or ice) for birthing and nursing their young [1], [2]. Many pinnipeds also mate on land and most prefer to be on a solid substrate when they undergo their annual moult (when seals replace their hair and several layers of skin) 2–4. Outside the breeding and moulting periods, some pinnipeds, such as hooded seals (*Cystophora cristata*) and Ross seals (*Ommatophoca rossii*), undertake long migrations and remain pelagic for months at a time without hauling out to rest on ice or land [5], [6]. However, other species remain more sedentary throughout the year and perform haul-out behaviour quite routinely [7], [8]. Hauling out of the water has clear thermoregulatory advantages under the right (warm, calm weather) environmental conditions [2], and during the annual moulting period being in the air allows seals to circulate blood to their skin without undue heat loss to the water, which permits fast and energetically efficient hair re-growth [9], [10]. Pinnipeds also haul out to rest and sleep away from the risk of aquatic predators [2], [11], minimizing the risk of terrestrial predation by using solid substrates close to quick aquatic escape routes. Hauling out in groups that share predator vigilance also provides advantages to pinnipeds in terms of the opportunity to rest more deeply while minimizing predation risk [2], [12].

Harbour seals are a coastal species that hauls out regularly in modestly tight social groups along coastlines throughout much of the north temperate region in both the Pacific and Atlantic Oceans [2], [13]. The haul-out behaviour of this species has been studied intensively in some parts of the species' range. General patterns include seasonal, circadian and tidal rhythms in the number of animals ashore as well as marked preferences being displayed for warm, dry weather [7], [14]. However, these patterns vary between different geographical areas and seasons, probably in accordance with regional and seasonal dietary variation as well as differing demands in the various phases of their life cycle [7], [15], [16]. Harbour seals spend a lot of time hauled out during the pupping, nursing and moulting periods in summer [14], [15]. During autumn they are more aquatic, foraging heavily to regain their energy stores [14], [17]. Age and maturity status as well as sex are also known to influence haul-out behaviour patterns [16], [18]. Harbour seals generally exhibit strong fidelity to haul-out locations [19], [20]. But, they do switch their preferred haul-out locations over the course of the year in response to factors such as seasonally available foraging opportunities, movement to breeding areas and avoidance of areas with heavy ice cover [16], [21], [22].

**Figure 1:**
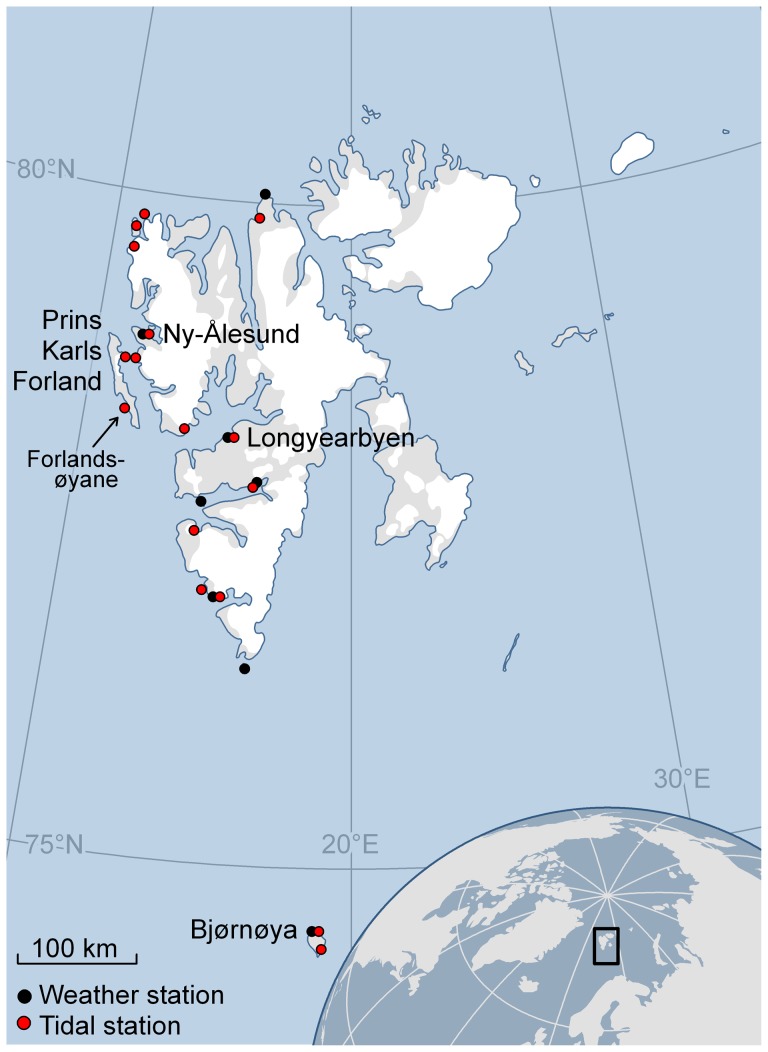
Map of Svalbard, Norway. Map of Svalbard, Norway, showing the location of Prins Karls Forland, the core area for the harbour seal population, and Forlandsøyane, the major capture area for the 60 harbour seals equipped with Satellite-Relay Data Loggers (SRDLs) in 2009 and 2010. The red and black dots show the location of the tidal and weather stations, respectively, that were used in the analyses of haul-out behaviour.

The harbour seal population residing at Svalbard, Norway, represents the northernmost population of this largely temperate species [23], [24]. The core of the harbour seals' range in Svalbard is around Prins Karls Forland, a 90 km long island on the west coast of Svalbard (Figure 1), which is the only area where pupping has been recorded within the Archipelago [25]. The Svalbard population of harbour seals is highly genetically distinct and has limited gene flow with neighbouring populations [26]; it comprises close to 2000 individuals (1888±1660-3023; [27]).

Being the world's northernmost population of this species, Svalbard harbour seals experience extreme light, ice and temperature regimes compared with other places in this species' range. Presumably in response to these conditions, Svalbard harbour seals have become shorter and proportionally heavier than conspecifics in more southerly locations, indicating that they carry a greater blubber mass and thus have a reduced lower critical temperature [28]. Contrary to other populations [29], the harbour seals in Svalbard exhibit marked sexual dimorphism, with mature males being both longer and heavier than mature females [28]. Svalbard harbour seals also have reduced longevity compared to more southerly populations, with the harsh environmental conditions they experience likely being a contributing factor [28]. Polar night, when the sun does not come above the horizon, lasts from approximately the middle of October to the middle of February, and the seals experience average winter temperatures close to −20°C (with record cold of −43.7°C, Norwegian Meteorological Institute, www.met.no).

Most terrestrial harbour seal haul-out sites are inaccessible during the winter period in Svalbard due to a combination of heavy seas causing icing of the shorelines and concentrating drift ice along the shores, in addition to the formation of some shore-fast ice in areas sheltered by islands or shallow shelves. Harbour seals populations in some regions display strong avoidance of ice [22], [30], while other populations readily use this substrate for hauling out [31], [32]. However, this species is not generally able to maintain breathing holes in ice and thus their winter distribution is limited to areas with open water or very loose drift ice [33]. How Svalbard's harbour seals respond to the arctic winter conditions is currently unknown.

This study uses Satellite-Relay Data Loggers (SRDLs) to perform an in-depth study of haul-out behaviour of the harbour seal population on Svalbard, focusing on the winter period (at-sea behaviour will be reported elsewhere). The goals of this study were: 1) to quantify haul-out behaviour throughout the year; 2) to explore the potential use of ice as a haul-out platform; 3) to compare the winter haul-out behaviour of seals in Svalbard to temperate populations in order to explore whether arctic winter conditions might serve as a long-term stressor that currently limits longevity and survivorship in this population and; 4) to relate the behaviour of Svalbard's harbour seals to the extreme environmental conditions experienced by this “temperate seal” in its northernmost population in order to explore the extent of their behavioural plasticity and likely responses to climate change.

## Materials and Methods

### Animal ethics statement

All research activities conducted during this study were carried out under permits from the Norwegian Animal Care Authority (Forsøksdyrutvalget Ref. 2009/1449) and the Governor of Svalbard (Sysselmannen på Svalbard Ref. 2009/00103-2 a.512) and followed best practice for all animal handling [34].

### Capture

Harbour seals were live-captured on the west coast of Prins Karls Forland, Norway, mainly around Forlandsøyane (Figure 1), during the summers and autumns of 2009 and 2010. Thirty pups (summer) and 30 immature/mature seals (autumn) were captured during the study period, 15 of each age group each year. The pups were captured toward the end of their nursing period from 29 June to 02 July, 2009 and from 02–05 July, 2010. The immature and mature seals were captured at the end of their annual moult from 01–13 September, 2009 and from 23 August to 03 September, 2010. Pups were captured in the water using hoop-nets from an inflatable boat (see [35]) while immature and mature seals were caught using tangle nets set close to shore near haul-out sites (see [36]). After being captured all seals were transferred to individual restraint-nets where they were weighed (Salter spring scales ±0.5 kg) and sex was determined. Immature and mature seals were immobilized with an intramuscular injection of Telazol at a dose of 1 mg/kg for immature seals and mature females and 0.75 mg/kg for mature males, before a lower incisor was extracted for age determination. Age was determined by counting the number of cementum growth layers in the decalcified, stained longitudinal sections of the extracted incisors [37]. While the immature and mature seals were immobilized, standard length and girth were measured to the nearest centimetre [38]. Pups were simply physically restrained to attach instruments; morphological measurements were not attempted.

Conductivity-Temperature-Depth – Satellite-Relay Data Loggers (CTD-SRDLs) (Sea Mammal Research Unit (SMRU) instrumentation, University of St Andrews, St Andrews, Scotland) were glued to the hair on the back of immature and mature seals mid-dorsally using quick-setting epoxy (tag mass 545 g, average 1.0% (range: 0.7–1.3%) of seal body mass). Pups were similarly equipped, but with a lighter, standard SMRU SRDL (tag mass 370 g, average 1.6% (range: 1.4–2.1%) of seal body mass). Both types of SRDLs have a wet-dry switch that documents when an animal is hauled out, in addition to other sensors (see http://www.smru.st-andrews.ac.uk/Instrumentation/Products/). Data collected by the SRDLs were transmitted via the ARGOS system (Advanced Research and Global Observation Satellite System, Toulouse, France; see [39], [40]).

### Data preparation

A haul-out event was defined as any period in which the wet/dry sensor on the SRDL was dry for ≥10 min and a haul-out period ended when the sensor was wet for at least 40 s. Two consecutive haul-out events that had end-start times less than three min apart were combined and treated as a single haul-out event. Each haul-out event is given a sequential number by the SRDL which made it possible to determine what percentage of haul-out events were transmitted by the ARGOS system. Missing haul-out events were “filled in” when possible, using the data summary chart generated by the tag, which records what percentage of time a seal spends hauled out, diving and swimming at the surface during a specified time period, as well as location data. These filled-in haul-out events were removed from analyses involving haul-out duration because the precision of the duration values is less certain for some of these events.

Haul-out locations were calculated by SMRU. SMRU filters the transmitted locations through a speed filter [41], which removes locations that would require an unrealistic swimming speed. Locations transmitted during the course of a haul-out event were averaged to compute the haul-out location. If no locations were transmitted during a given haul-out interval, the location was estimated via linear interpolation along the track line for the animal based on time.

Sex and maturity status (i.e. pup, immature and mature, see [28]), as well as environmental, spatial and temporal variables, were included as covariates in statistical models exploring haul-out behaviour of the seals (Figure 1, Tables 1 and 2). Air pressure and change in air pressure (over 3, 12 and 24 hr periods) were included in the analyses as a proxy for wind because the weather stations on Svalbard are all located on shore, where wind speed and direction is very dependent on the topography in the vicinity of each station [42]. High pressures systems during the winter in Svalbard are often associated with calm fair weather, clear skies and cold temperatures, whereas low pressure systems are more often associated with warmer temperatures, but overcast conditions and stronger winds. [42], [43].

**Table 1:** Factor variables.

Variable	Factor level	Description	Resolution	Source
Light*	0	dark: sun elev <−12	minute	Astronomical Applications Department of the US Naval Observatory (http://aa.usno.navy.mil/)
	1	light: sun elev >0	as above	as above
	2	nautical dawn: −12< sun elev <0 in the morning	as above	as above
	3	nautical dusk: −12< sun elev <0 in the evening	as above	as above
Substrate	0	land	1 km	Norwegian Meteorological Institute (http://polarview.met.no)
	1	shore-fast ice	as above	as above
	2	off-shore ice	as above	as above
	3	floating pieces of glacier ice	as above	as above
Year	1	seals tagged in 2009		this study
	2	seals tagged in 2010		as above
Sex	M	male		this study
	F	female		as above
Maturity**	P	pup: age 0		this study
	I	immature: age 1–2		as above
	M	mature: age 6+		as above

* inputs of 78.5°N and 11.0°E were used.

** seals aged 3–5 were classified based on their length, girth and body measurements as per [28].

Description of the factor variables, including an explanation of the different factor levels, their resolution and source, used in the analyses of haul-out behaviour for the 60 harbour seals equipped with Satellite-Relay Data Loggers (SRDLs) in Svalbard, Norway in 2009 and 2010.

**Table 2:** Continuous variables.

Variable	Abbreviation	Units	Resolution	Source
Air temperature	temp	°C	hour	Norwegian Meteorological Institute (http://eklima.met.no)
Time to and from low tide	tide	minute	minute	Norwegian Mapping Authority (http://www.statkart.no)
Fraction of the moon illuminated	lunar	percent	day	Astronomical Applications Department of the US Naval Observatory (http://aa.usno.navy.mil/)
Air pressure (at sea level)	pres	hPa	hour	Norwegian Meteorological Institute (http://eklima.met.no)
Absolute air pressure change over last 3, 12, 24 hrs	pres3, pres12, pres24		hour	Norwegian Meteorological Institute (http://eklima.met.no)
Solar hour	shour	hour	hour	local2Solar function in solaR library in R.2.15.2
Month	month		month	this study
Time to previous haul-out event	lasth	atomic time	minute	this study
Latitude	lat	degree	>250 m	this study
Longitude	lon	degree	>250 m	this study

Description of the continuous variables, including their abbreviation, units, resolution and source, used in the analyses of haul-out behaviour for the 60 harbour seals equipped with Satellite-Relay Data Loggers (SRDLs) in Svalbard, Norway in 2009 and 2010.

ArcMap10 (ESRI, Redlands, CA, USA) was used to find the closest tide and temperature station for each of the seals' transmitted locations and also to explore the relationship between haul-out locations and sea ice distribution (Table 1). Fine-scale errors associated with ARGOS locations and the ice shapefiles undoubtedly results in some degree of misclassification between land and shore-fast ice. Month was used as a proxy for seasonal changes in haul-out behaviour. Ice and light regimes vary widely over the year, and breaking the year into monthly periods created time frames containing roughly similar environmental conditions.

### Haul-out behaviour variables and modelling

Haul-out behaviour was examined from September to June. Large scale patterns such as haul-out platform use or length of aquatic periods between haul-out events were explored on monthly time scales, whereas haul-out probabilities and haul-out durations with respect to weather variables etc. were explored on hourly time scales (based on the resolution of data collection).

Data exploration was carried out as per [44], [45]. Generalized Additive Mixed Models (GAMMs) were used in these analyses as they allowed the non-linear relationships identified during data exploration to be modelled by smooth functions [46]. Continuous variables were standardized for use in statistical analyses. All statistical analyses were completed in R 2.15.2 [47] and Akaike Information Criterion corrected for small samples size (AICc; [48]) was calculated using the MuMIn package [49]. Models were selected based on their AICc as recommended in [48].

Results are presented as mean ±95% confidence intervals (CI), except where otherwise indicated. CIs for haul-out duration, time between haul-out events and the use of off-shore ice as a haul-out platform (by maturity group and year) were calculated by bootstrapping (1000 repetitions) using the boot package in R [50] because they were not normally distributed. The jack.after.boot function in the boot package was used to assess the influence that each seal had on the bootstrap results. All maps were made using ArcMap10 (ESRI, Redlands, CA, USA).

### Time between haul-out events and use of off-shore ice as a haul-out platform

The time since a haul-out event (*y*) and the use of off-shore ice as a haul-out platform (*y*) for the *i* ^th^ seal for the *j^t^* ^h^ time was modelled by: 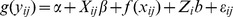


Where *g* represents a link function for the response variable, *X* represents a fixed effect, *β* a parameter vector for the fixed effect, *f* a smooth factor for the *x* variable, *Z* an independent random effect for the *i* ^th^ seal and *b* a vector of the coefficients for the random effect. Fixed covariates included month, maturity, sex and year of tagging. Seal id was included as a random effect using the random intercept method [44]. Month was included as a cubic regression spline; k  =  8 was chosen so that the shape of the covariate function could be accurately deduced [46]. For the model analysing the time between haul-out events an identity link was used for the log-transformed response variable; the Gaussian family was used to assess the residual variance. For the model analysing the use of off-shore ice as a haul-out platform a logistic link was use for the response variable; the binomial family was used to assess the residual variance. The gamm4 package [51] was used to do these analyses. The gam.check function was used to verify model fit and the deviance and normalized residuals were plotted against the explanatory covariates included and excluded from the selected models. For the use of off-shore ice as a haul-out platform model the residuals, fitted and raw values were grouped by seal id and month to verify model fit. The smooth term from the AICc selected models was applied to the deviance residuals with an increased k to ensure that there was no remaining pattern that could be explained by a higher k value. A quantile-quantile plot was used to check for linearity of the random effects and ANOVAs of the normalized residuals versus the fitted values were conducted to test for any remaining structure.

### Haul-out probability

Each seals' data record was divided into half-hour intervals and an interval was given a value of 1 if the seal was hauled out during the majority of that time interval and a 0 if it was not. A GAMM that incorporated temporal autocorrelation was used to analyse the haul-out probability using the mgcv package in R [46]. The response variable was included in the model with a logistic link; the binomial family was used to explore residual variance. All covariates and interactions of interest were included in a model that was run for each month from September to June (light was removed from the models for the months of midnight sun (May and June)), so that the effects of each covariate on haul-out probability and how the effects varied over the course of the year could be explored [52]. The full model is: 
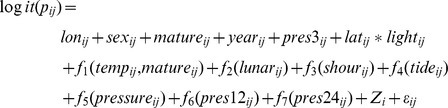



Where *p_ij_* is a probability parameter denoting a binary outcome for the *i^th^* seal at the *j^th^* time, *Z* is a random effect of seal id and *ε* is a temporally autocorrelated random effect used to account for repeated observations from a seal not being independent. Temporal autocorrelation for each seal was modelled using the autoregressive model of order 1 (corAR1) structure [44], with seal id included as a grouping factor.

Temperature, lunar, tide, pressure, pres12 (12 hr interval) and pres24 (24 hr interval) were smoothed with a cubic regression spline and solar hour was added as a cyclic cubic regression spline smooth. k was decreased, or the covariate was entered into the model linearly in some months to achieve convergence. Model validation was carried out for each month in the same way as for the off-shore ice model.

### Haul-out duration

The Cox Proportional-Hazards regression model (CPH; [53]) was used to assess the relationship between haul-out duration and the covariates listed above using the survival package in R [54]. The hazard function represents the risk of ending a haul-out event at time *t*, given that this has not already occurred. The hazard function (λ) for observation (i.e. haul-out event) *i* is, 
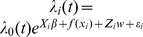
 where *X* represents the fixed covariates for haul-out event *i*, *β* a vector of the coefficients, *f* a penalized spline smoothing function of covariate *x*, *w* a vector containing the unknown random effects (frailties) for each seal, *Z* a design matrix, equalling 1 if a haul-out event *i* is performed by seal *j* and 0 if the situation is otherwise, and *λ_0_* the baseline hazard, which is a positive function of time. The hazard ratio for two observations (i.e. haul-out events) *i* and *k* is, 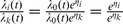 making it independent of time [55]. A hazard ratio above 1 indicates an increased risk of ending a haul-out event while a hazard ratio below 1 indicates a decreased risk of ending a haul-out event.

One model was run for each month to assess the varying impact of the covariates on haul-out duration throughout the study period. The covariates included were the same as for the haul-out probability models, with the addition of haul-out substrate and time since the previous haul-out event. The log of time since the previous haul-out event, tide and solar hour were included as penalized spline smoothing functions (psplines) with df = 4. Seal id was included as a shared gamma frailty term with a theta of 0.03 (the variance of the frailty term in the model with all the haul-out data) [55]. The theta for frailty was set to account for numerical problems encountered in the estimation of frailty in some months (indicated by zero variance estimates). All ten monthly models were investigated for model fit. The cox.zph function [55] using the scaled Schoenfeld residuals was used to test the validity of the proportional hazards assumption. The presence of influential observations was checked for using dfbeta residuals and martingale residuals were used to check for non-linearity of covariates. [55], [56].

## Results

### Satellite tag performance

**Figure 2:**
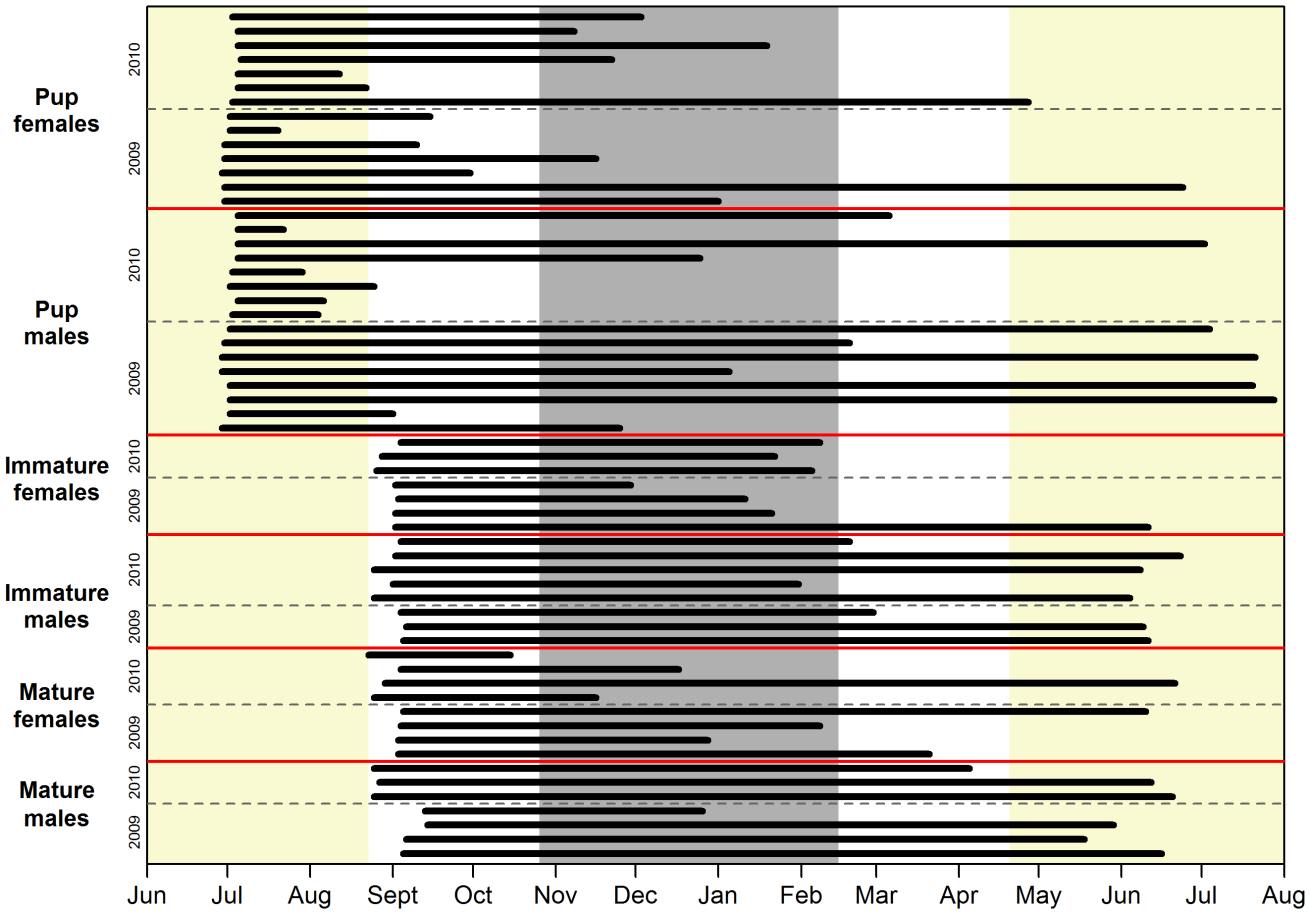
Tag life. Duration of data records (“tag life”) for 60 harbour seals equipped with Satellite-Relay Data Loggers (SRDLs) in Svalbard, Norway in 2009 and 2010. The grey rectangle represents the time period covered by the polar night (sun beneath the horizon), the yellow rectangles represent time periods covered by midnight sun and the white rectangles are light transition periods that include both night and daylight.

The average duration of the 60 harbour seal data records was 188±106 days (mean ± sd), but there was considerable variation among individuals (range = 20–393 days; Figure 2, Tables S1, S2). Male seals had a longer tag life than females (221 days (CI: 181–261 days) vs 153 days (CI: 122–185 days), ANOVA, F = 7.11, p = 0.01). Tag life did not differ according to the year of tagging or maturity status of the seals, though the extremes at both ends were displayed by pups. Eight of the 9 seals that transmitted data for less than two months and the five seals that transmitted data for over a year were all pups (Table S1).

Data records contained an average of 178±85 haul-out events per seal (mean ± sd, min = 50, max = 426). Pups (1.5 haul-out events/day (CI: 1.2–1.7 haul-out events/day)) hauled out more often than immature (0.9 haul-out events/day (CI: 0.8–1.0 haul-out events/day), ANOVA, F = 19.04, p<0.0001) or mature seals (0.8 haul-out events/day (CI: 0.7–0.9 haul-out events/day), ANOVA, F = 25.29, p<0.0001), while no difference existed between immature and mature seals (ANOVA, F = 0.33, p = 0.5672; Tables S1, S2). The overall average percentage of haul-out events that were transmitted was 92±8% (mean ± sd). Adding untransmitted haul-out events, when these could be calculated from summary statistics produced by the tags or sequential haul-out numbers, brought the total coverage to 99±5% (mean ± sd; Tables S1, S2) of the complete data records. All pup records were complete (Table S1).

### Seasonality in haul-out durations and time since the previous haul-out event

**Figure 3:**
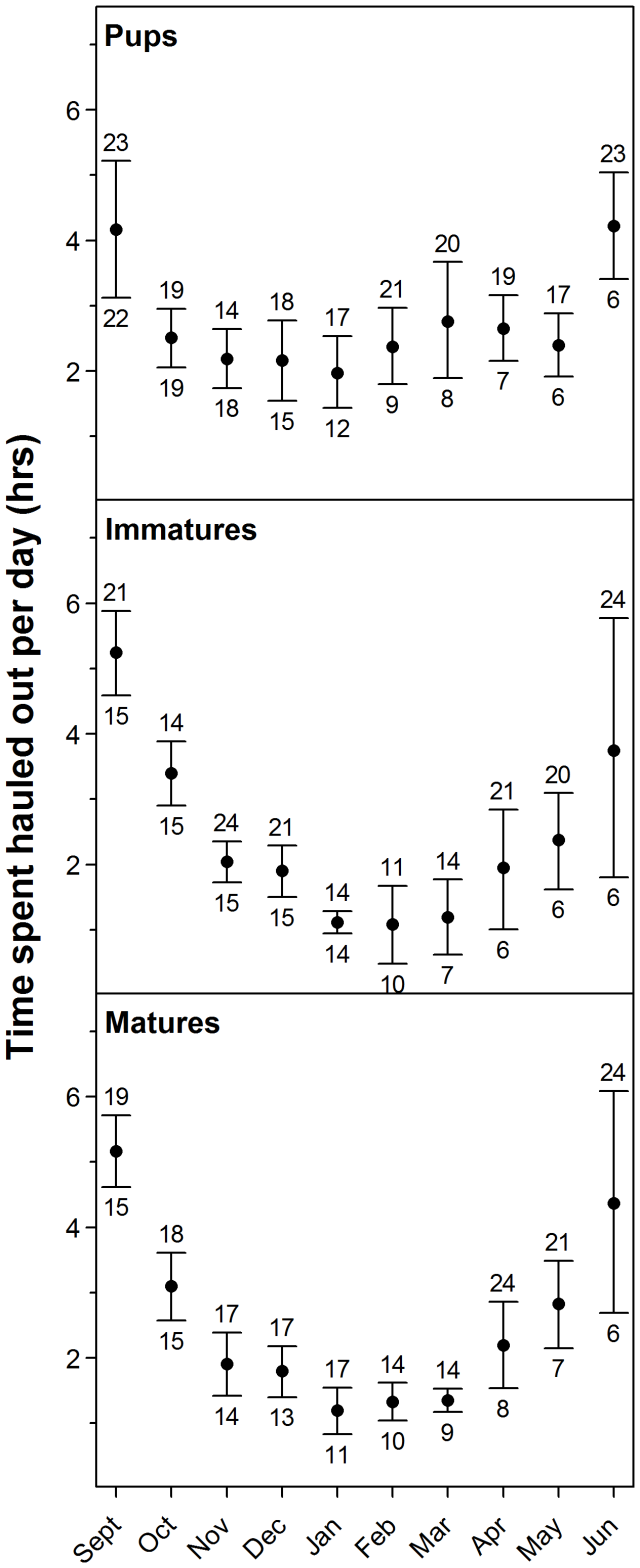
Number of hours spent hauled out each day. Mean daily number of hours (±95% CI) 60 harbour seals equipped with Satellite-Relay Data Loggers (SRDLs) in Svalbard, Norway in 2009 and 2010, spent hauled out by season (month) and age group (pups, immature and mature seals). The number at the top of the CI bar indicates the maximum amount of time (hrs) an individual seal spent hauled out in that month and the number at the bottom of the CI bar indicates the number of seals transmitting data in that month.

The average and maximum haul-out durations were 3.6 hrs (CI: 3.4–3.8 hrs) and 42.3 hrs, respectively. The average time between haul-out events was 26 hrs (CI: 24–29 hrs) despite most (73%) aquatic periods being less than 24 hrs. The maximum aquatic period, i.e. period between haul-out events was 19 days. The mean (±95% bootstrapped CI) amount of time spent hauled out per day was calculated for each maturity group by month. Pups spent an average of 2.4 hrs/day (10% of their time) hauled out and this proportion stayed quite constant over the duration of the study period, except for September and June when they hauled out more (4.2 hrs/day, 18% of their time; Figure 3). For immature and mature seals, the average number of hours spent hauled out decreased from 5.2 hrs/day (22% of their time) in September to 1.2 hrs/day (5% of their time) in February and subsequently increased again to 4 hrs/day (17% of their time) in June (Figure 3). The maximum time spent hauled out daily for an individual seal ranged between 11 and 24 hrs (46–100% of their time, on a monthly basis). There was a seasonal trend, which reached a minimum in February (Figure 3). The influence exerted by individual variation was in general minor.

**Figure 4:**
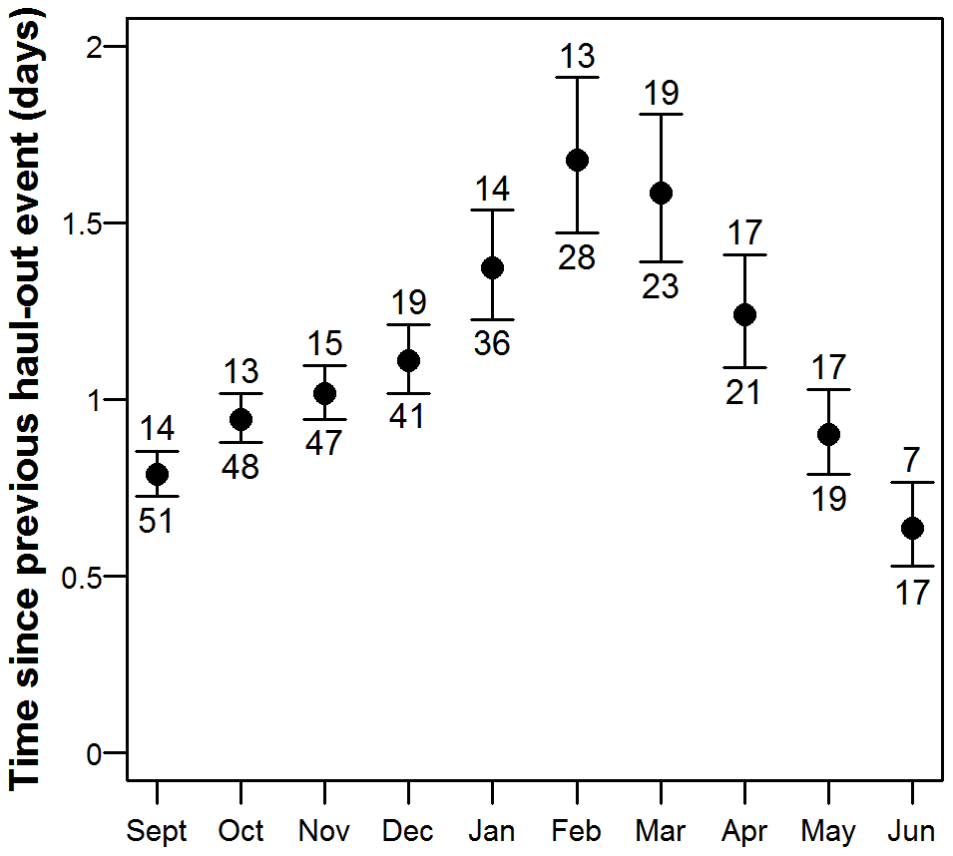
Time since previous haul-out event. Output from a GAMM model showing the mean number of days (±95% CI) since the previous haul-out period, plotted by month for the 60 harbour seals equipped with Satellite-Relay Data Loggers (SRDLs) in Svalbard, Norway in 2009 and 2010. The number at the top of the CI bar indicates the maximum time between haul-out events (days) for an individual seal and the number at the bottom of the CI bar indicates the number of seals transmitting data in that month.

The best model describing time since the previous haul-out event contained the variable month (Table S3). No structure remained in the normalized residuals (F = 0.13, p = 0.721). The number of days since the previous haul-out event increased from less than one day in September to over 1.5 days in February and decreased once again to less than one day in June (Figure 4). The maximum time spent in the water (i.e. without hauling out) by individual seals ranged between 13–19 days across the months, except in June when the longest aquatic period was only 7 days (Figure 4). Four of the monthly maximums (September, October, November and April) were performed by pups, three (March, May and June) by immature seals and three (December, January and February) by mature seals. The December and January maxima were done by the same mature male (M56-09) and the September and November maxima were done by the same male pup (M25b-09).

### Haul-out substrates

**Figure 5:**
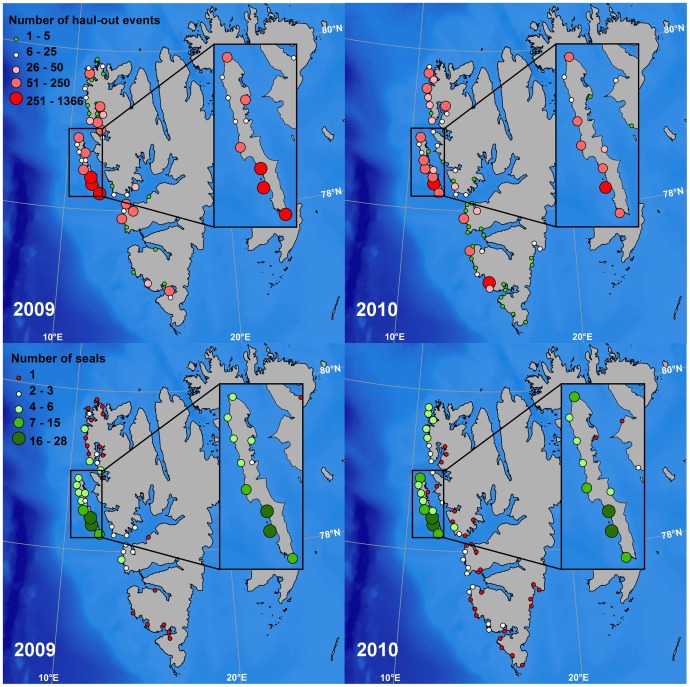
Terrestrial haul-out site locations and the use of specific sites by individual harbour seals. Maps showing the location of terrestrial haul-out events performed by 60 harbour seals equipped with Satellite-Relay Data Loggers (SRDLs) (top) and the number of individual harbour seals using specific terrestrial haul-out sites (bottom) (30 tagged each year) in 2009 and 2010 in Svalbard, Norway. The island in the inset map is Prins Karls Forland, the principle breeding site and core distribution area for harbour seals in Svalbard.

The average haul-out duration was similar between the four substrate categories (land, 3.8 hrs (CI: 3.5–4.1 hrs); shore-fast ice, 3.4 hrs (CI: 3.2–3.7 hrs); off-shore ice, 3.5 hrs (CI: 3.0–3.9 hrs); floating glacier ice (including haul-out events in August), 2.8 hrs (CI: 1.8–3.9 hrs)). The number of terrestrial haul-out events was similar between the two years. Most seals used terrestrial haul-out sites around Prins Karls Forland (their capture location), with other sites being used by only a few individuals (Figure 5). Of the 45 terrestrial haul-out locations used by only one seal, 38 were used by pups, 5 by immature seals and 2 by mature seals.

**Figure 6:**
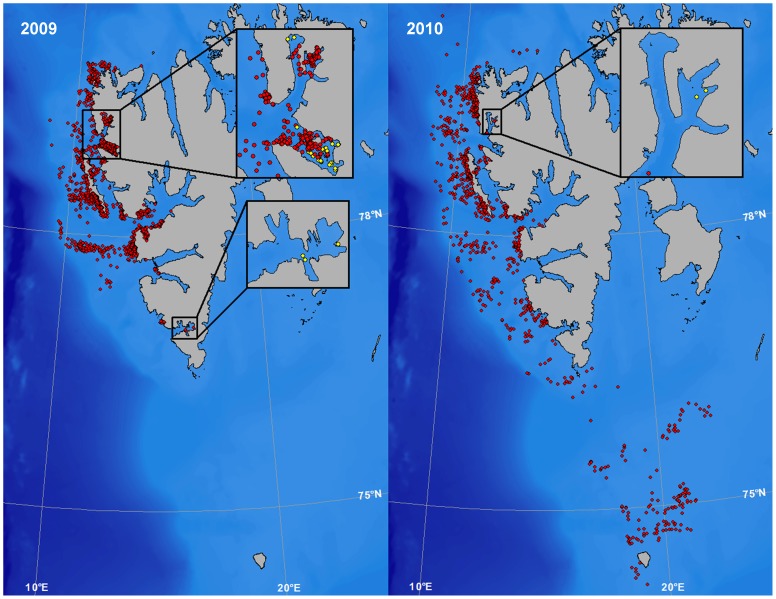
Non-terrestrial haul-out sites. Maps showing the location of non-terrestrial haul-out events performed by 60 harbour seals equipped with Satellite-Relay Data Loggers (SRDLs) in 2009 and 2010 in Svalbard, Norway. The seals hauled out on either shore-fast ice, drifting pack-ice or on floating glacier ice pieces (yellow dots in inset maps) in fjords, when annually formed sea ice was not available.

The locations of non-terrestrial haul-out sites (shore-fast ice, off-shore ice and glacier ice) were markedly different between the two years, with the seals using a larger overall area in 2010 compared to 2009. Four pups travelled south towards Bjørnøya in 2010 (Figure 6). Thirty-one haul-out events were identified to have taken place on floating pieces of glacier ice in the two years of the study (Figure 6). The majority of these haul-out events took place in August, while some few took place in September, October and November. All of the haul-out events on glacier ice were undertaken by pups.

**Figure 7:**
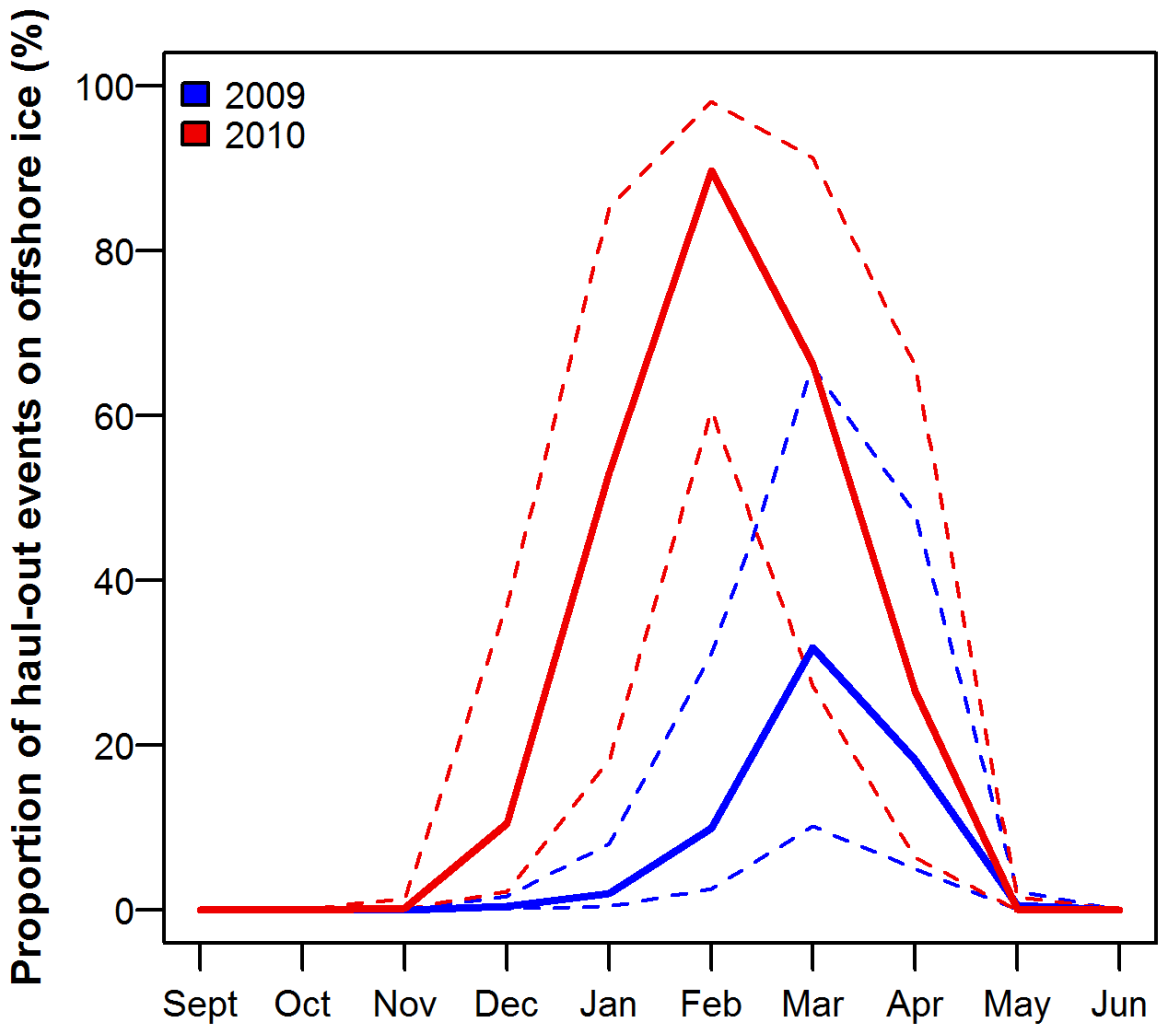
Proportion of haul-out events on off-shore ice. Smooth curves from a GAMM model, showing the proportion of haul-out events (mean (solid lines) ±95% CI (dotted lines)) performed by 60 harbour seals, equipped with Satellite-Relay Data Loggers (SRDLs) in Svalbard, Norway, which took place on off-shore ice, by month during 2009 and 2010.

The best model describing the use of off-shore ice as a haul-out platform included maturity and an interaction between month and year (Table S4). No structure remained in the normalized residuals (F = 0.47, p = 0.493). The seals in the second year of tagging (2010) utilized off-shore ice to a greater extent than in the first year of the study (2009). This difference was most apparent in winter (November to February) with the use of off-shore ice in spring (April to May) being more similar between the two years (Figure 7, Table S5). The use of off-shore ice was dependent on the maturity status of the individual, with pups tending to use it more as a haul-out platform than the immature seals (t = −2.00, p = 0.0452; Table S5). There was no significant difference in the use of off-shore ice between pups and mature seals (t = −1.72, p = 0.0860) or between immature and mature seals (t = 0.31, p = 0.7543; Table S5). Individual variability did exist in the use of off-shore ice. Seal ID had the greatest jackknifed influence on the bootstrapped confidence intervals early and late in the sea ice season, when the use of off-shore ice by each individual seal, compared to the rest of the seals in the tagging year and month, depended highly on their location.

### Factors affecting haul-out probability and haul-out duration

**Figure 8:**
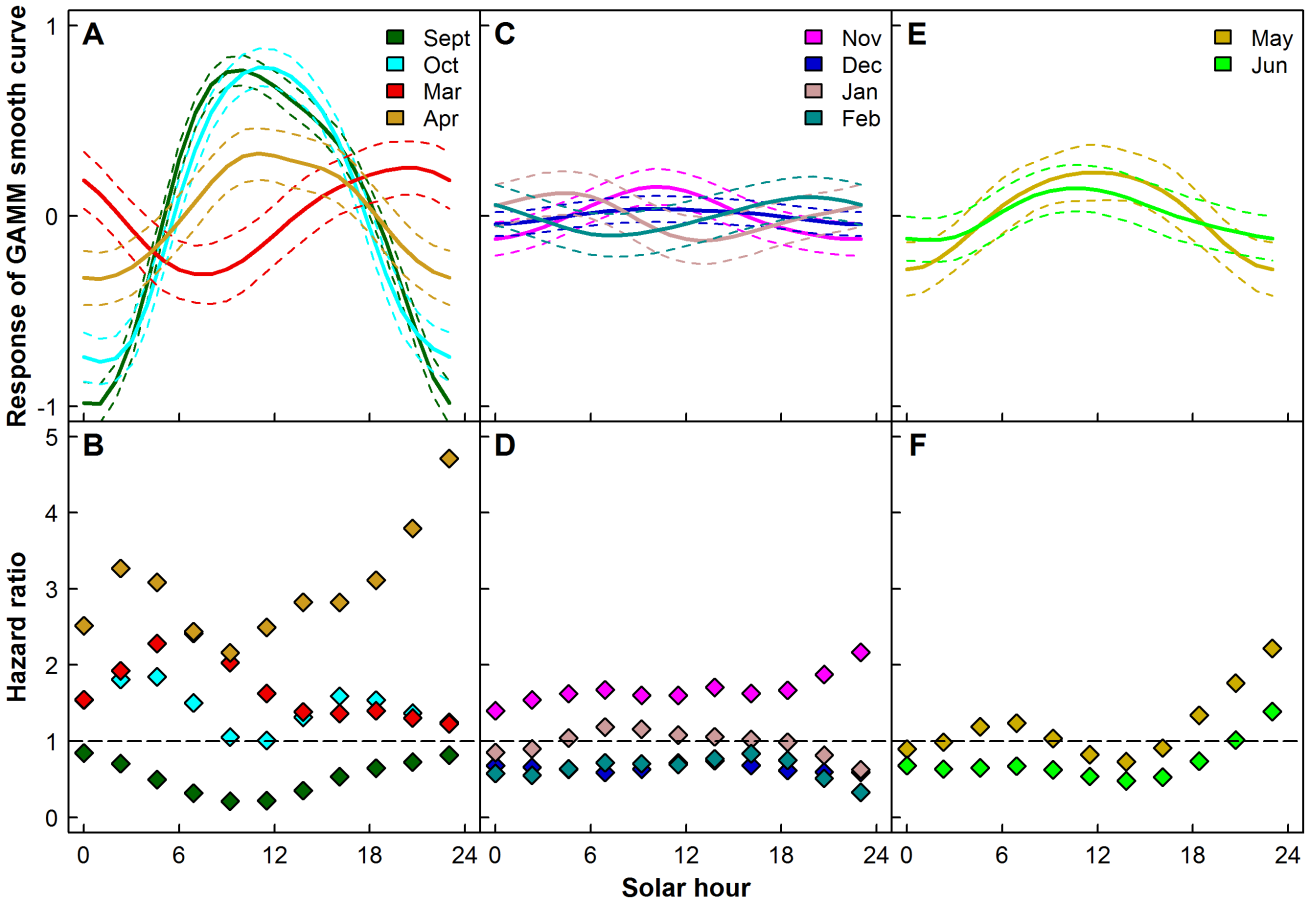
Haul-out behaviour indices vs solar hour. GAMM smooth curves (A, C, E - mean (solid lines) ±95% CI (dashed lines)) showing impacts of solar hour on haul-out probability and hazard ratios from Cox Proportional Hazard (CPH) models (B, D, F) for the months where there is a day and night cycle (A,B), for the months of polar night (C,D) and for the months of midnight sun (E,F) for 60 harbour seals equipped with Satellite-Relay Data Loggers (SRDLs) in 2009 and 2010 in Svalbard, Norway. [Hazard ratios beneath one indicate a decreased risk of ending a haul-out event and hazard ratios above one indicate an increased risk of ending a haul-out event.]

The model parameters and statistics for the haul-out probability and haul-out duration analyses and the results of the ANOVAs between the normalized residuals and fitted values can be found in Tables S6 and S7. The probability of hauling out peaked at midday during the months of the year that had a daily rhythm of light and dark (September, October, March and April) with the exception of March (Figure 8). The risk of ending a haul-out event generally decreased during these months from midnight until noon and increased thereafter (Figure 8). In March, the highest probability of hauling out occurred around midnight, and the risk of ending a haul-out event was the lowest in the afternoon and did not increase again until after midnight. During the months of polar night (November, December, January and February) haul-out probability and the risk of ending a haul-out event were more or less constant across the 24 hr cycle (Figure 8). In May and June, the months of midnight sun, haul-out probability was slightly elevated around solar noon compared to the remainder of the day. The risk of ending a haul-out event was relatively constant but increased somewhat after 14:00 (Figure 8).

Seals had a higher probability of hauling out in daylight or at nautical dawn (sun less than 12 degrees below the horizon) during September and October, compared to darkness (sun more than 12 degrees below the horizon), and had a decreased probability of ending a haul-out event in nautical dawn and dusk compared to darkness. There was a preference to haul out, and a decreased risk of ending a haul-out event, at nautical dawn and dusk compared to darkness in November and January (when there was nautical dawn and dusk but not daylight). In February (polar night ends February 15) and March, this pattern was reversed. The probability of hauling out in daylight, nautical dawn and dusk decreased compared to darkness and the risk of ending a haul-out event increased during these time periods compared to darkness.

**Figure 9:**
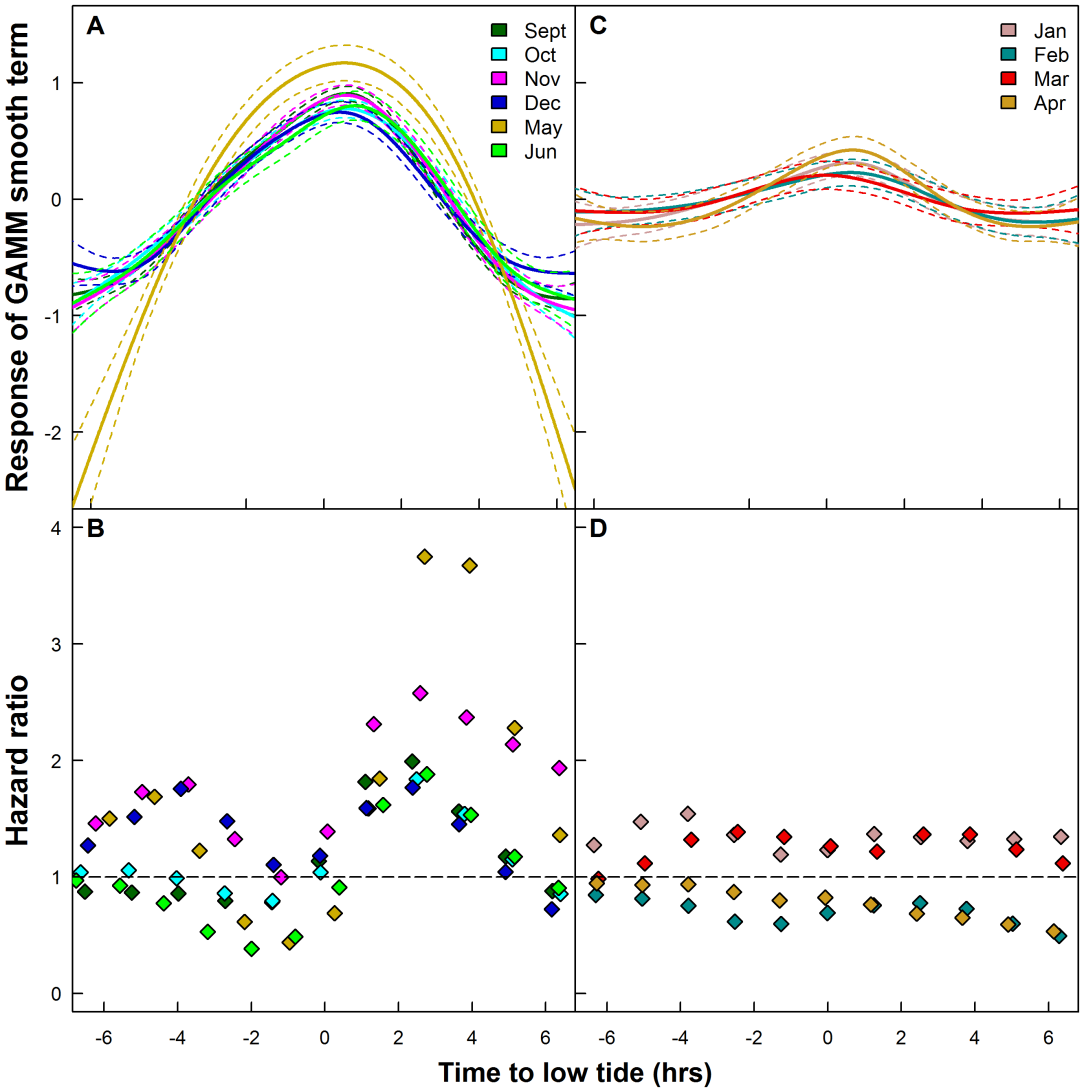
Haul-out behaviour indices vs tide. GAMM smooth curve (A, C, - mean (solid lines) ±95% CI (dashed lines)) showing impacts of the time to low tide (hrs) on haul-out probability and hazard ratios from Cox Proportional Hazard (CPH) models (B,D) for the months where the primary haul-out substrate is land (A,B) and the months where the primary haul-out substrate is sea ice (C,D) for the 60 harbour seals equipped with Satellite-Relay Data Loggers (SRDLs) in 2009 and 2010 in Svalbard, Norway. [Hazard ratios beneath one indicate a decreased risk of ending a haul-out event and hazard ratios above one indicate an increased risk of ending a haul-out event.]

Haul-out probability and the risk of ending a haul-out event in relation to tidal patterns varied over the course of the study period. During the months where the primary haul-out platform was land (September, October, November, December, May and June), the probability of being hauled out increased towards low tide and decreased thereafter (Figure 9). The risk of ending a haul-out event also decreased in the hours leading up to low tide and then increased thereafter. For most of the aforementioned months, there was also a decrease in the risk of ending a haul-out event towards high tide (Figure 9). In the months where the primary haul-out platform was ice (January, February, March and April), the seals still showed a preference to haul out around low tide, but the magnitude of the trend was greatly reduced (Figure 9). There was little change in the risk of ending a haul-out event over the tidal cycle during these months (Figure 9).

A decreased probability of hauling out was observed as temperature increased for all age classes in most months in the study period (Figures S1, S2); concomitantly, there was also an increased risk of ending a haul-out event as temperature increased. But, there was an increased probability of hauling out as air pressure (at sea level) increased (Figure S3) and a decreased probability of hauling out as absolute pressure change over the last 3 hrs increased for most months. There was no consistent relationship between haul-out duration and air pressure or change in air pressure.

Mature and immature seals showed similar haul-out probabilities and risks of ending haul-out events during most months, with a few notable exceptions. Mature seals had a higher probability of hauling out and a decreased risk of ending a haul-out event compared to immature seals in February and May. Pups generally had an increased probability of hauling out and a decreased risk of ending their haul-out events compared to the other age classes for most of the study period. Sex also had an influence on haul-out behaviour. Males had a higher probability of hauling out and a decreased risk of ending a haul-out event compared to females in February, March and May.

Haul-out substrate influenced the risk of ending a haul-out event. Seals using shore-fast ice or off-shore ice had a decreased risk of ending a haul-out event compared to seals on land in November and May (when all substrates were available). Glacier ice pieces were not used often enough to explore details regarding patterns of use.

Year of tagging and latitude did impact haul-out behaviour but not in a consistent manner. Longitude affected haul-out probability, with an increased probability of hauling out as the seals moved east. The effect of the fraction of moon illuminated was highly variable among months with no general trend emerging. The time between haul-out events did not affect haul-out duration.

## Discussion

This study is the first to investigate the haul-out behaviour of the harbour seal population within the High Arctic Archipelago of Svalbard, Norway during the arctic winter. Approximately one-third (n = 19) of the 60 tags deployed in this study sent data until the subsequent moulting period. A likely explanation for the failure of some tags is breakage of the antennae; many of the tags stopped transmitting, or began to have large gaps in the transmitted data, at a time that coincided with the onset of the sea ice season. Data records for males were longer than for females, possibly because of some sex-specific behaviour that makes females in this population more prone to break their tags' antennae, though it is not obvious what such behaviour might involve. The failure rate for tags deployed on pups is likely linked to pup mortality which is estimated to be anywhere from 20–65% in this species [17], [57], [58], while older age classes have a lower annual mortality of ∼20% [57], [58]. Some pups in this study definitely died over the course of the year. In both 2009 and 2010, polar bears (*Ursus maritimus*) were observed killing harbour seal pups, including an instrumented individual, on Prins Karls Forland (CL and KMK, pers. obs.). Additionally, several pups abruptly ceased transmitting data when they were far offshore in open water with no sea ice. Greenland sharks (*Somniosus microcephalus*) and killer whales (*Orcinus orca*) are both potential aquatic predators of harbour seals in Svalbard waters [59], [60]. The lack of an age effect on tag life was surprising given the different mortality risks among age classes.

Many studies have deployed satellite-linked tags or VHF radio tags on harbour seals but only a few studies have focused on time periods outside breeding and moulting (i.e. [20], [22], [61]) and even fewer have looked specifically at haul-out behaviour outside these periods (i.e. [14], [16], [62]). Harbour seals in Svalbard spend less time hauled out in winter and autumn (September/October) compared to other populations although their general haul-out behaviour patterns are similar [63]. In Svalbard, immature and mature seals spent on average 5.2 hrs/day (22% of a day) hauled out during the early autumn and this decreased to 1.2 hrs/day (5% of a day) in the winter months. In more southerly populations, these age groups haul out ∼25–30% of the time in the early autumn and ∼11–18% in the winter [7], [16], [64].

The thermoneutral zone in harbour seals is governed by the thickness of their blubber layer. Blubber thickness of harbour seals is at its minimum during the breeding and moulting periods and at its maximum in the winter months following intensive feeding in autumn [17], [65]. Lower critical temperature of this species has been measured to be 10°C in water (based on juveniles weighing from 26–50 kg; [66], [67]), and 3°C for pups [68], −2.3°C for juveniles [69] and below −10°C for immature and mature seals in air [66], [70]. But, the harbour seals in Svalbard are both shorter and fatter than conspecifics in more southern populations, and thus have a thicker blubber layer [28]. Model calculations suggest that a change in blubber thickness of 10% can change the lower critical temperature by almost 10°C [67]. But, even with their reduced lower critical temperature, hauling out during winter in Svalbard might expose them to conditions where they are thermally stressed. Resting in the water where temperatures can only be as low as about −1.8°C, as opposed to land where temperatures can be ≤−40°C, is likely often thermally advantageous in winter.

Seasonal variation in the number of hours hauled out was dampened in Svalbard pups compared to other age classes, with time spent hauled out being almost constant from October to May. Pups hauled out less during the early autumn and spring and more during the winter compared to older seals. This could be due to pups' larger surface to volume ratio. The thermal conductivity of water is 20 times higher than that of air, so staying in the water may be disadvantageous for pups at temperatures where the older age groups may cope more easily. Other explanations might include a greater need to escape aquatic predators while sleeping because their small size may make them more vulnerable, or a greater need to circulate blood to their skin given their higher growth rates compared to older age classes.

The time between haul-out events changed seasonally in a manner similar to harbour seals in other populations, such as in California, where the seals haul out a lot during breeding and moulting but less (only 50% of the days) outside these periods [71]. The maximum aquatic periods in Svalbard for each month (13–19 days September to May, 7 days in June) were much longer than what has been found for other populations [7], [14], [16], though the average aquatic period for Svalbard harbour seals was similar to the average for more southern populations [16], [20], [64].

The dominant terrestrial haul-out area in this study was the Prins Karls Forland area on the west coast of Spitsbergen [72], [73]. But, some few seals also used terrestrial haul-out sites on the west and south coasts of Spitsbergen. Pups accounted for most of the haul-out locations that were only visited by one individual and they also travelled to the most distant areas. Harbour seal pups are known to exhibit large dispersal movements compared to older age classes, especially during the first months after weaning [21], [74]. They are presumably learning how and where to find food, which is not necessary for older, more experienced, individuals.

Haul-out events on shore-fast and off-shore ice took place predominantly in the waters west of the main terrestrial haul-out areas. Harbour seals cannot maintain breathing holes in heavy shore-fast ice [33] and are known to avoid areas with extensive ice cover in some parts of their range [22], [30]. Shore-fast ice is very limited in any case along the west coast of Svalbard because of the influence of the West Spitsbergen Current (WSC), a branch of the North Atlantic Current, which transports warm, saline Atlantic water along the western coast of Spitsbergen [75], [76]. The WSC creates warmer temperatures along the west coast compared to the east coast of Svalbard, as well as other areas at this latitude. The effects of the WSC likely explain the presence of harbour seals at this high northerly latitude in Svalbard. However, drift ice can be extensive along the coast in some years [77], [78] and heavy seas cause icing on the shore, which in combination makes most of the terrestrial harbour seal haul-out sites inaccessible during the winter period in Svalbard.

The tagged seals had a much broader winter distribution in 2010–2011 compared to 2009–2010, corresponding to much earlier ice formation and much more extensive ice cover in the second year of the study (Norwegian Meteorological Institute, http://met.no). The seals used off-shore ice as a haul-out platform earlier and more extensively in 2010–2011. The pups used off-shore ice to a greater extent than the older seals early in the season in both years, suggesting that they more actively avoid swimming through areas with extensive ice cover. The use of ice as a haul-out platform in Svalbard might actually have some energetic advantages. In the months where both ice and land were available as haul-out platforms, the seals had longer haul-out durations when they were on ice. They might save transport costs by hauling out near where they are feeding. Another factor that might influence the choice of a particular substrate for hauling out is differential availability based on tides. Seals hauling out in intertidal areas on islands have to wait for the site to become available, whereas seals hauling out on ice can haul out whenever they choose.

In Alaska, harbour seals use glacier ice extensively, as a pupping and moulting platform as well as for resting [31], [32]. But, this is certainly not the case in Svalbard where these non-resting activities take place on land; harbour seals are rarely reported from the areas in front of calving tidal glaciers in Svalbard. In this study only pups used glacier ice for hauling out with certainty, though its use by immature and mature seals cannot be completely excluded because some use of glacier ice pieces might be masked by ARGOS position errors.

When hauling out on land, the harbour seals in Svalbard show a strong preference to haul out around low tide (this study, [36], [62]), similar to many other populations [14], [15] because low tide exposes their favoured inter-tidal areas. When using ice as a haul-out platform, the association between haul-out behaviour and tide decreased, although a slight trend persisted. Tidal state has been found to be of little importance when harbour seals haul out on glacier ice elsewhere [79] or more generally in areas where tide does not dictate the availability of their haul-out sites [80], [81].

Harbour seals prefer to haul out at midday in many areas [15], [19], including Svalbard, during the months where there was day night cycling and also during periods with midnight sun (this study, [36], [62]). But in Svalbard, the seals preferred to haul out in the darkest part of the day in February and March. A shift in the prey that the seals are targeting or a shift in the behaviour of the prey are potential reasons for this switch in behaviour. Harbour seals in Scotland switch their prime time for feeding to daytime during winter because their prey are more accessible during that day when the fish cluster in dense schools in trenches and holes [82].

There was no pattern between haul-out behaviour of Svalbard harbour seals and solar hour during the period of the polar night. Similar to terrestrial animals on Svalbard, such as Svalbard reindeer (*Rangifer tarandus platyrhynchus*) [83] and Svalbard rock ptarmigan (*Lagopus mutus hyperboreus*) [84], the seals ceased having circadian rhythms during the polar night. But during this period, there was a tendency for the seals to haul out during nautical dawn and dusk. They might be better able to detect approaching terrestrial predators with even this small amount of available light. Other studies have found that outside of the breeding and moulting periods, when the primary activity is foraging, harbour seals generally decrease the diurnal patterning of their activity, even in areas where there is a strong day/night cycle [16], [71], though some studies report that a diurnal cycle is maintained, though its amplitude decreases [63].

The relationship between temperature and haul-out probability and duration in the Svalbard harbour seal population was opposite to that found in other regions, where harbour seals prefer to haul out when it is warmer [13], [15]. This seems counterintuitive given that one would expect arctic harbour seals to take advantage of any available heat. But, in the winter in Svalbard warmer temperatures are often associated with low pressure systems and stormy conditions while colder temperatures are often associated with high pressure systems and fair weather [42]. Wind speed and wind chill are both factors that have negative influence on harbour seal haul-out behaviour [15], [85], and these factors apparently override absolute temperature effects in Svalbard in winter. High wind speeds also lead to increased wave intensity which might cause water spray problems when they are on ice, which would be chilling and perhaps also compromise their view of their surroundings. Air pressure and change in air pressure were therefore included as proxies for wind speed in this study and haul-out probability did increase with increasing air pressure and decrease relative to increasing changes in air pressure (over relatively short periods).

In February, March and May, male seals had a higher probability of hauling out than female seals. Previous research in western Scotland has also suggested that females spend less time hauled out than males outside the breeding and moulting periods [16]. Females have higher reproductive costs than males [2] and may have to forage more during the rest of the year to replenish body stores. But, some caution is warranted in over-interpreting these results because the sex ratio of the seals transmitting data during the winter was not balanced in this study (February, 17 males were transmitting data compared to only 6 females).

During the winter the immature and mature seals behaved quite similarly in terms of their haul-out patterns. But, towards spring mature seals were out of the water more. Pregnant females are approaching birthing and mature males may be moving from foraging areas into regions where they will establish themselves for breeding [86]. Moonlight intensity was included in this study as previous research has indicated a potential lunar influence on harbour seal haul-out behaviour [7], [15], and on the behaviour of other pinnipeds such as Galapagos fur seals (*Arctocephalus galapagoensis*) [87]. However, it did not impact the haul-out behaviour of the harbour seals in Svalbard.

## Conclusion

Svalbard harbour seals currently display many similarities with southern counterparts regarding their haul-out behaviour, but they haul out significantly less during winter compared to other populations (except for pups), and Svalbard harbour seals exhibit extensive use of sea ice during the winter, when tidal and diel rhythms are dampened greatly or lost entirely. Pups appear to be unable to spend extended periods in the water, perhaps because of their small body size; the resultant exposure to environmental extremes might lower their survival rate. Climate change is projected to continue the warming trend and ice reductions of the past decades in the Arctic and harbour seals are likely to benefit from these future changes [88]; alterations of their haul-out behaviour and a more normal survivorship and longevity might thus be expected in Svalbard harbour seals some decades into the future.

## Supporting Information

Figure S1 **Haul-out behaviour index vs temperature (September-January).** GAMM smooth curves (mean (solid lines) ±95% CI (dashed lines)) showing the impacts of temperature (°C) on haul-out probability from September to January for the 60 harbour seals equipped with Satellite-Relay Data Loggers (SRDLs) in Svalbard, Norway in 2009 and 2010. Pups are in the left column (red), immature seals are in the middle column (blue) and mature seals are in the right columns (grey).
(TIF)

Figure S2 **Haul-out behaviour index vs temperature (February-June).** GAMM smooth curves (mean (solid lines) ±95% CI (dashed lines)) showing the impacts of temperature (°C) on haul-out probability from February to June for the 60 harbour seals equipped with Satellite-Relay Data Loggers (SRDLs) in Svalbard, Norway in 2009 and 2010. Pups are in the left column (red), immature seals are in the middle column (blue) and mature seals are in the right columns (grey).
(TIF)

Figure S3 **Haul-out behaviour index vs air pressure at sea level.** GAMM smooth curves (mean (solid lines) ±95% CI (dashed lines)) showing the impacts of air pressure at sea level (hPa) on haul-out probability for the 60 harbour seals equipped with Satellite-Relay Data Loggers (SRDLs) in 2009 and 2010 in Svalbard, Norway, by month.
(TIF)

Table S1 Tag performance statistics for the pups. Tag statistics for harbour seal pups equipped with Satellite-Relay Data Loggers (SRDLs) in Svalbard, Norway in 2009 and 2010. The first letter of the seal ID indicate the sex, the numbers before the dash indicates the pup's body mass at the time of capture (kg), and the number after the dash indicate the year in which they were tagged.
(DOCX)

Table S2 Tag performance statistics for the immature and mature seals. Tag statistics for immature and mature harbour seals equipped with Conductivity-Temperature-Depth – Satellite-Relay Data Loggers (CTD-SRDLs) in Svalbard, Norway in 2009 and 2010. The first letter of the seal ID indicates the sex, the numbers before the dash indicate the seals' body mass at the time of capture (kg) and the numbers after the dash indicate the year in which they were tagged.
(DOCX)

Table S3 AICc table for time since previous haul-out event. The corrected Akaike information criterion (AICc), change in AICc and weight of the AICc for the different GAMM models for the time since previous haul-out event for the 60 harbour seals equipped with Satellite-Relay Data Loggers (SRDLs) in Svalbard, Norway in 2009 and 2010. Ytag is the year of tagging and maturity indicates whether the seal was a pup, immature or mature.
(DOCX)

Table S4 AICc table for use of off-shore ice as a haul-out platform. The corrected Akaike information criterion (AICc), change in AICc and weight of the AICc for the different GAMM models for the use of off-shore ice as a haul-out platform by the 60 harbour seals equipped with Satellite-Relay Data Loggers (SRLDs) in Svalbard, Norway in 2009 and 2010. Ytag is the year of tagging and maturity indicates whether the seal was a pup, immature or mature.
(DOCX)

Table S5 Percentage of haul-out events on off-shore ice. Percentage of haul-out events each month that took place on off-shore ice (mean ± bootstrapped 95% CI) for each maturity group by year for the 60 harbour seals equipped with Satellite-Relay Data Loggers (SRDLs) in Svalbard, Norway in 2009 and 2010.
(DOCX)

Table S6 Haul-out probability. GAMM model results for haul-out probability for the 60 harbour seals equipped with Satellite-Relay Data Loggers (SRDLs) in Svalbard, Norway in 2009 and 2010, showing the estimated degrees of freedom (edf) and p-values for the smooth terms, the variance of the random effect and the value of temporal autocorrelation (phi). The estimate (est), 95% CI and p-values are provided for the linear terms. Values of 0.5 indicate a 50/50 chance of hauling out vs not hauling out, with values beneath 0.5 indicating a greater probability of not hauling out and values over 0.5 indicating a greater probably of hauling out. The reference levels are: maturity = immature seals; sex = female; year of tagging (ytag) = 2009 (the first year of tagging) and; light = total darkness (sun <12 degrees below horizon), except where stated otherwise.
(DOCX)

Table S7 Haul-out duration. Cox Proportional Hazard model results for haul-out duration for the 60 harbour seals equipped with Satellite-Relay Data Loggers (SRDLs) in Svalbard, Norway in 2009 and 2010, showing the hazard ratio (exp(coef)), 95% CI and the p-values for each covariate. For the smooth terms only the p-values are given. The reference levels are: maturity = immature seals; sex = female; year of tagging (ytag) = 2009 (first year of tagging); light = total darkness (sun <12 degrees below horizon), except where stated otherwise and; haul-out substrate = land, except where stated otherwise.
(DOCX)
