# Supplementary figures and images for: Haul-Out Behaviour of the World's Northernmost Population of Harbour Seals (Phoca vitulina) throughout the Year
Source: PLoS One. 2014 Jan 22;9(1):e86055. doi: 10.1371/journal.pone.0086055 (PMC3899210; doi:10.1371/journal.pone.0086055)

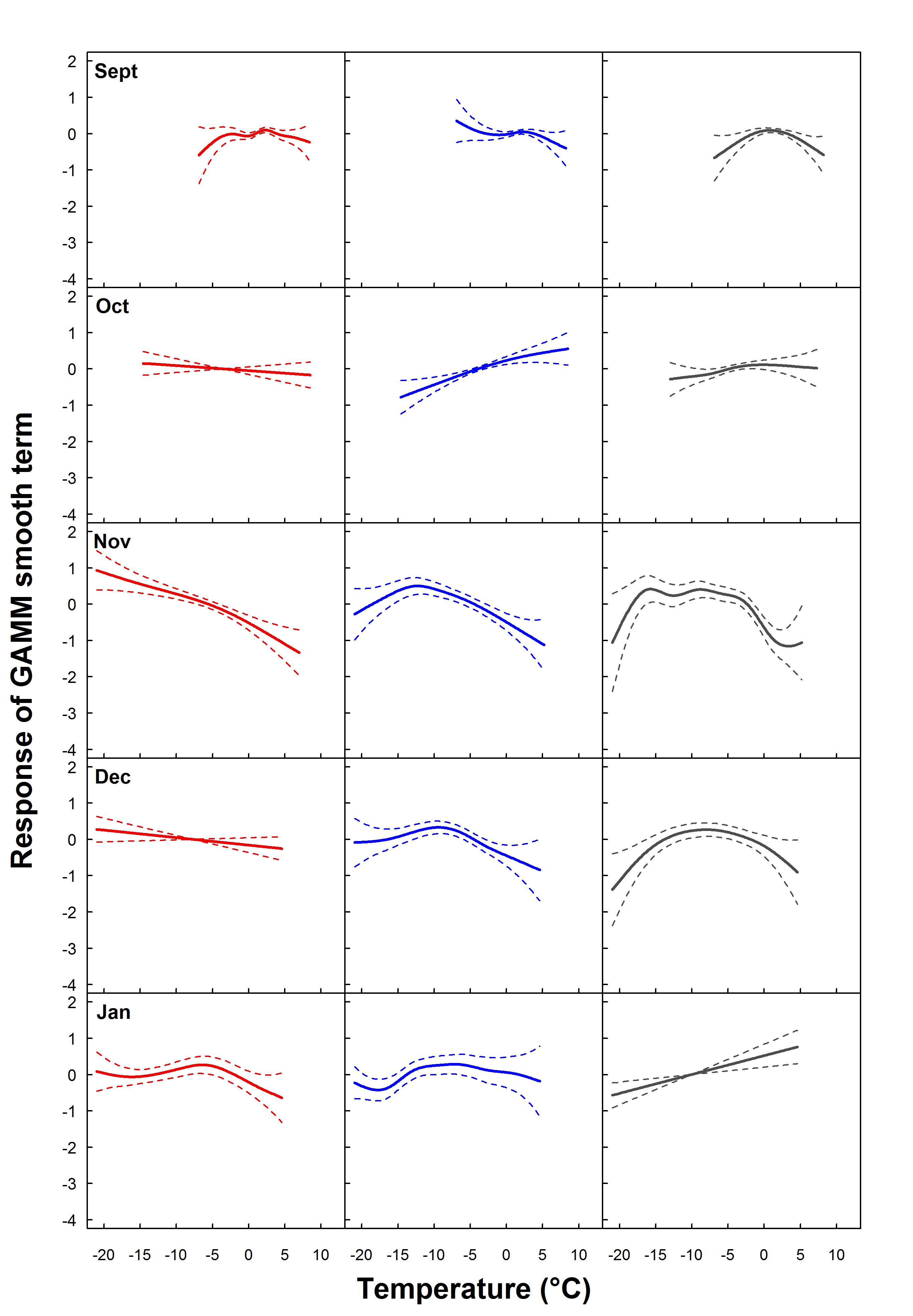

Supplement: Figure S1 — Haul-out behaviour index vs temperature (September-January). GAMM smooth curves (mean (solid lines) ±95% CI (dashed lines)) showing the impacts of temperature (°C) on haul-out probability from September to January for the 60 harbour seals equipped with Satellite-Relay Data Loggers (SRDLs) in Svalbard, Norway in 2009 and 2010. Pups are in the left column (red), immature seals are in the middle column (blue) and mature seals are in the right columns (grey). (TIF) [file pone.0086055.s001.tif]

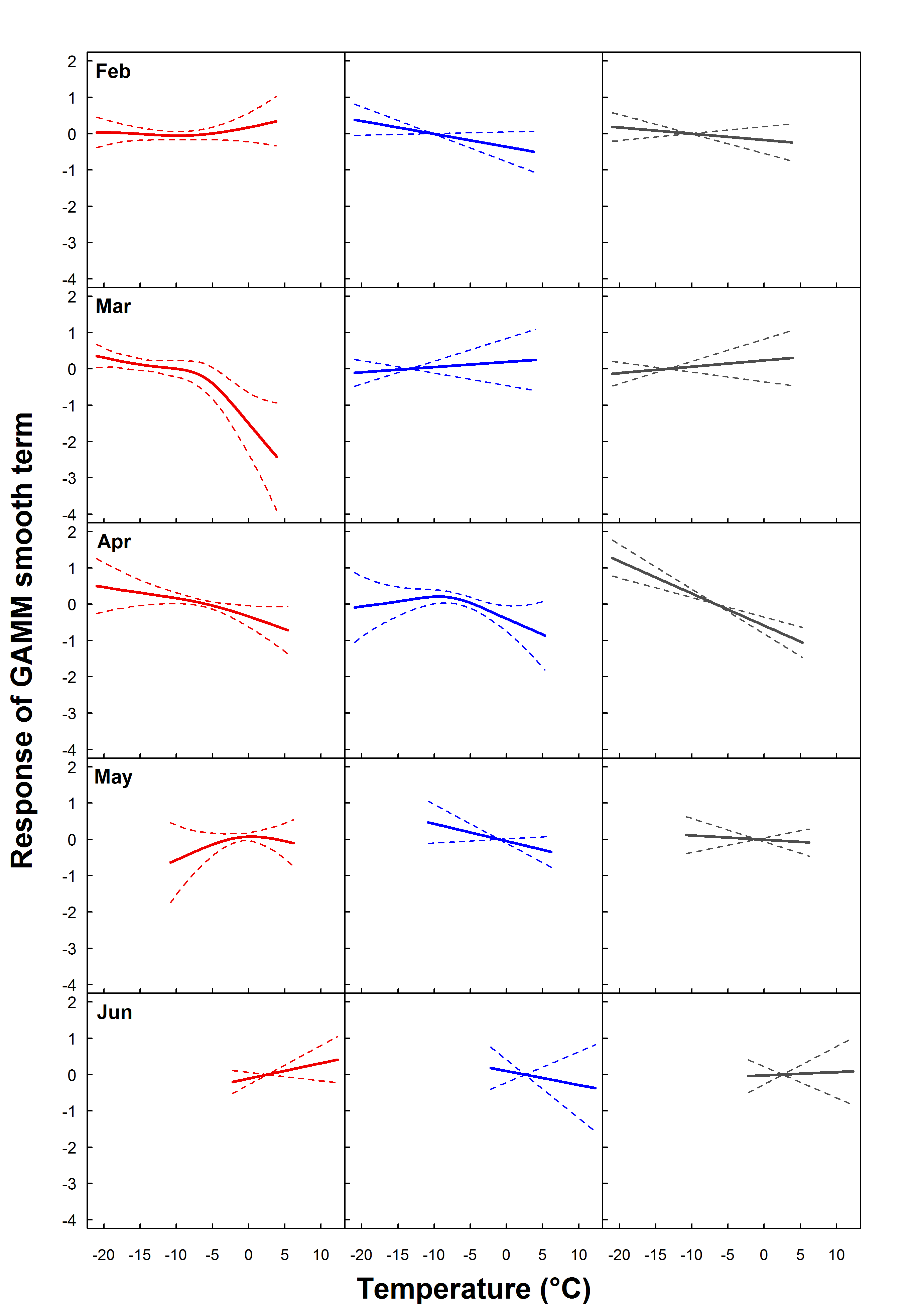

Supplement: Figure S2 — Haul-out behaviour index vs temperature (February-June). GAMM smooth curves (mean (solid lines) ±95% CI (dashed lines)) showing the impacts of temperature (°C) on haul-out probability from February to June for the 60 harbour seals equipped with Satellite-Relay Data Loggers (SRDLs) in Svalbard, Norway in 2009 and 2010. Pups are in the left column (red), immature seals are in the middle column (blue) and mature seals are in the right columns (grey). (TIF) [file pone.0086055.s002.tif]

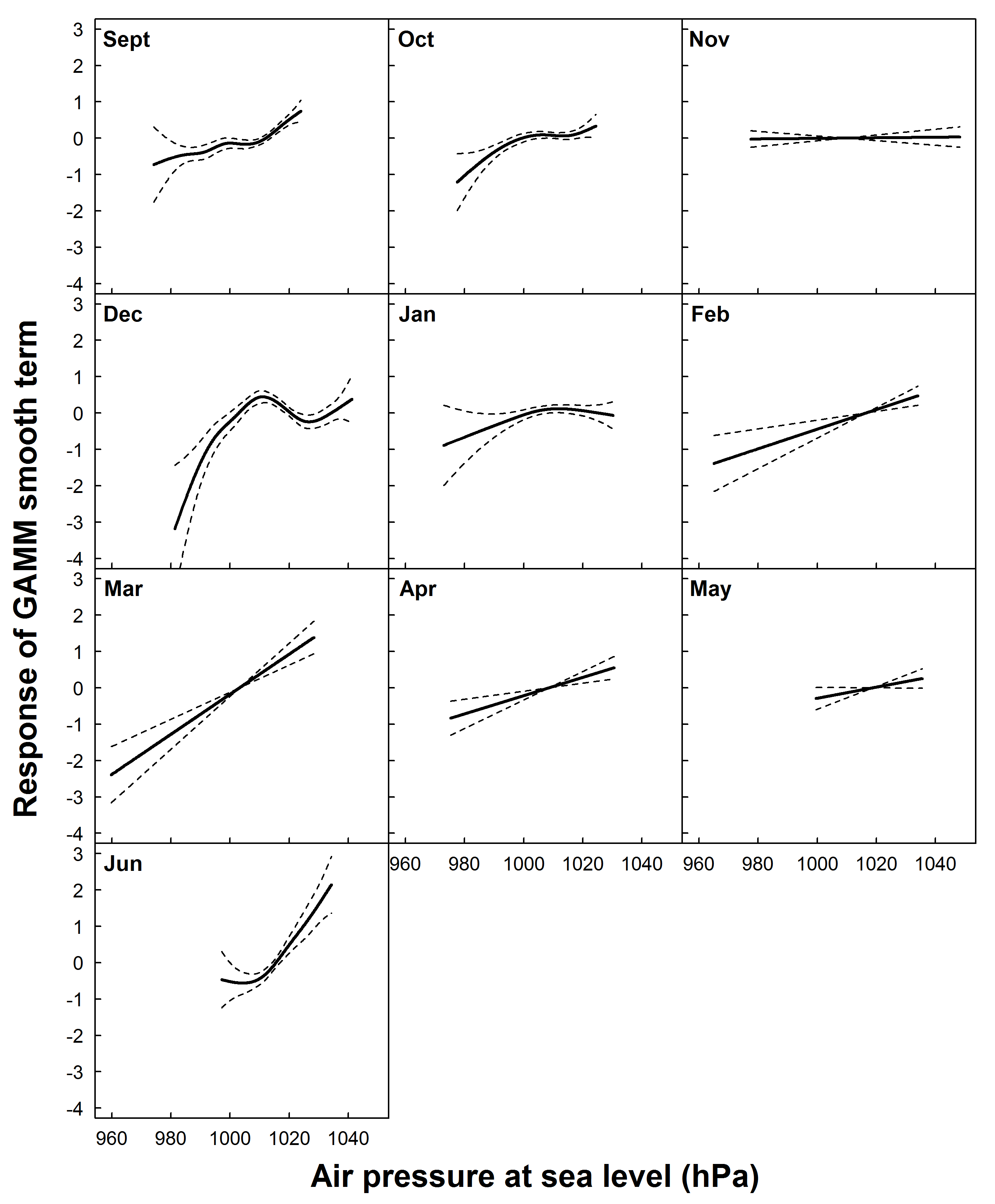

Supplement: Figure S3 — Haul-out behaviour index vs air pressure at sea level. GAMM smooth curves (mean (solid lines) ±95% CI (dashed lines)) showing the impacts of air pressure at sea level (hPa) on haul-out probability for the 60 harbour seals equipped with Satellite-Relay Data Loggers (SRDLs) in 2009 and 2010 in Svalbard, Norway, by month. (TIF) [file pone.0086055.s003.tif]
