# Supplementary material for: Haul-Out Behaviour of the World's Northernmost Population of Harbour Seals (Phoca vitulina) throughout the Year
Source: PLoS One. 2014 Jan 22;9(1):e86055. doi: 10.1371/journal.pone.0086055 (PMC3899210; doi:10.1371/journal.pone.0086055)
Supplement: Table S1 — Tag performance statistics for the pups. Tag statistics for harbour seal pups equipped with Satellite-Relay Data Loggers (SRDLs) in Svalbard, Norway in 2009 and 2010. The first letter of the seal ID indicate the sex, the numbers before the dash indicates the pup's body mass at the time of capture (kg), and the number after the dash indicate the year in which they were tagged. (DOCX) [file pone.0086055.s004.docx]

| **Seal ID** | **Date deployed** | **Tag life (d)** | **Number of haul-out events transmitted** | **Percentage of total haul-out events transmitted** | **Number of haul-out events added** | **Total percentage of haul-out event coverage** |
| --- | --- | --- | --- | --- | --- | --- |
| F20.5-09 | 30-06-2009 | 189 | 293 | 100 | 0 | 100 |
| F22a-09 | 29-06-2009 | 361 | 291 | 100 | 0 | 100 |
| F22b-09 | 29-06-2009 | 103 | 159 | 98 | 4 | 100 |
| F22c-09 | 30-06-2009 | 143 | 243 | 100 | 0 | 100 |
| F23-09 | 30-06-2009 | 73 | 158 | 99 | 2 | 100 |
| F26-09 | 02-07-2009 | 20 | 57 | 100 | 0 | 100 |
| F27.5-09 | 02-07-2009 | 67 | 90 | 100 | 0 | 100 |
| M22a-09 | 29-06-2009 | 157 | 200 | 100 | 0 | 100 |
| M22b-09 | 02-07-2009 | 62 | 83 | 100 | 0 | 100 |
| M23-09 | 02-09-2009 | 393 | 426 | 100 | 0 | 100 |
| M24.5-09 | 02-07-2009 | 384 | 365 | 100 | 0 | 100 |
| M24-09 | 29-06-2009 | 191 | 184 | 100 | 0 | 100 |
| M25a-09 | 29-06-2009 | 388 | 336 | 100 | 0 | 100 |
| M25b-09 | 30-06-2009 | 236 | 201 | 100 | 0 | 100 |
| M26-09 | 02-07-2009 | 371 | 418 | 100 | 0 | 100 |
| F19-10 | 03-07-2010 | 299 | 265 | 97 | 9 | 100 |
| F21.5-10 | 05-07-2010 | 49 | 87 | 100 | 0 | 100 |
| F21-10 | 05-07-2010 | 39 | 104 | 77 | 31 | 100 |
| F22-10 | 05-07-2010 | 146 | 197 | 99 | 2 | 100 |
| F25a-10 | 05-07-2010 | 205 | 171 | 98 | 4 | 100 |
| F25b-10 | 05-07-2010 | 141 | 152 | 99 | 1 | 100 |
| F26.5-10 | 03-07-2010 | 158 | 137 | 99 | 1 | 100 |
| M18-10 | 03-07-2010 | 33 | 90 | 88 | 12 | 100 |
| M20-10 | 05-07-2010 | 34 | 73 | 88 | 10 | 100 |
| M21.5a-10 | 02-07-2010 | 55 | 70 | 92 | 6 | 100 |
| M21.5b-10 | 03-07-2010 | 27 | 54 | 100 | 0 | 100 |
| M23-10 | 05-07-2010 | 176 | 204 | 99 | 3 | 100 |
| M24.5a-10 | 05-07-2010 | 367 | 258 | 97 | 8 | 100 |
| M24.5b-10 | 05-07-2010 | 28 | 44 | 88 | 6 | 100 |
| M27-10 | 05-07-2010 | 245 | 273 | 98 | 5 | 100 |
| **Mean ± SD** |  | 171 ± 127 | 189 ± 109 | 97 ± 5 | 3 ± 6 | 100 ± 0 |
