# Supplementary material for: Haul-Out Behaviour of the World's Northernmost Population of Harbour Seals (Phoca vitulina) throughout the Year
Source: PLoS One. 2014 Jan 22;9(1):e86055. doi: 10.1371/journal.pone.0086055 (PMC3899210; doi:10.1371/journal.pone.0086055)
Supplement: Table S2 — Tag performance statistics for the immature and mature seals. Tag statistics for immature and mature harbour seals equipped with Conductivity-Temperature-Depth – Satellite-Relay Data Loggers (CTD-SRDLs) in Svalbard, Norway in 2009 and 2010. The first letter of the seal ID indicates the sex, the numbers before the dash indicate the seals' body mass at the time of capture (kg) and the numbers after the dash indicate the year in which they were tagged. (DOCX) [file pone.0086055.s005.docx]

|  | **Seal ID** | **Date deployed** | **Tag life (d)** | **Age** | **Length (cm)** | **Girth (cm)** | | **Number of haul-out events transmitted** | **Percentage of total haul-out events transmitted** | **Number of haul-out events added** | **Total percentage of haul-out event coverage** |
| --- | --- | --- | --- | --- | --- | --- | --- | --- | --- | --- | --- |
| **Immature seals** | F41-09 | 01-09-2009 | 286 | 2 | 104 | | 90 | 174 | 72 | 65 | 97 |
|  | F44-09 | 01-09-2009 | 149 | 2 | 116 | | 89 | 136 | 78 | 39 | 100 |
|  | F47-09 | 02-09-2009 | 133 | 2 | 118 | | 92 | 139 | 86 | 22 | 100 |
|  | F48-09 | 01-09-2009 | 102 | 3 | 120 | | 93 | 79 | 82 | 17 | 100 |
|  | M43-09 | 03-09-2009 | 182 | 2 | 113 | | 86 | 148 | 86 | 25 | 100 |
|  | M51-09 | 04-09-2009 | 282 | 2 | 120 | | 96 | 196 | 96 | 9 | 100 |
|  | M52-09 | 05-09-2009 | 278 | 2 | 122 | | 98 | 183 | 91 | 18 | 100 |
|  | F42-10 | 26-08-2010 | 152 | 2 | 109 | | 88 | 123 | 78 | 32 | 94 |
|  | F44-10 | 28-08-2010 | 150 | 3 | 116 | | 88 | 124 | 95 | 6 | 100 |
|  | F53-10 | 03-09-2010 | 159 | 4 | 120 | | 92 | 96 | 83 | 18 | 95 |
|  | M41-10 | 24-08-2010 | 288 | 1 | 105 | | 89 | 150 | 93 | 11 | 100 |
|  | M45-10 | 31-08-2010 | 162 | 2 | 122 | | 86 | 111 | 80 | 27 | 96 |
|  | M48-10 | 24-08-2010 | 290 | 3 | 128 | | 86 | 178 | 86 | 27 | 90 |
|  | M53a-10 | 30-08-2010 | 298 | 3 | 126 | | 97 | 248 | 92 | 20 | 95 |
|  | M53b-10 | 03-09-2010 | 178 | 2 | 123 | | 93 | 122 | 90 | 10 | 71 |
| **Mean ± SD** |  |  | 206 ± 71 | 2 ± 1 | 117 ± 7 | | 91 ± 4 | 147 ± 43 | 86 ± 7 | 23 ± 14 | 96 ± 7 |
| **Mature seals** | F60-09 | 02-09-2009 | 203 | 4 | 126 | | 98 | 157 | 88 | 19 | 90 |
|  | F66-09 | 01-09-2009 | 126 | 5 | 133 | | 100 | 83 | 86 | 14 | 100 |
|  | F74-09 | 01-09-2009 | 164 | 11 | 139 | | 108 | 109 | 77 | 32 | 100 |
|  | F76-09 | 04-09-2009 | 282 | 9 | 139 | | 107 | 144 | 92 | 13 | 100 |
|  | M56-09 | 04-09-2009 | 287 | 4 | 128 | | 101 | 168 | 94 | 10 | 100 |
|  | M64-09 | 05-09-2009 | 257 | 3 | 130 | | 94 | 188 | 95 | 9 | 100 |
|  | M65-09 | 13-09-2009 | 264 | 3 | 128 | | 109 | 186 | 83 | 38 | 100 |
|  | M77-09 | 10-09-2009 | 110 | 5 | 142 | | 111 | 99 | 74 | 30 | 86 |
|  | F50-10 | 23-08-2010 | 55 | 3 | 127 | | 87 | 66 | 93 | 5 | 100 |
|  | F58a-10 | 25-08-2010 | 86 | 5 | 124 | | 95 | 68 | 94 | 4 | 100 |
|  | F58b-10 | 28-08-2010 | 300 | 5 | 132 | | 94 | 207 | 90 | 22 | 100 |
|  | F59-10 | 03-09-2010 | 106 | 3 | 125 | | 93 | 57 | 92 | 5 | 100 |
|  | M57-10 | 24-08-2010 | 302 | 5 | 132 | | 96 | 212 | 87 | 30 | 97 |
|  | M64-10 | 26-08-2010 | 292 | 4 | 127 | | 104 | 199 | 98 | 4 | 100 |
|  | M65-10 | 24-08-2010 | 236 | 5 | 137 | | 95 | 157 | 95 | 8 | 100 |
| **Mean ± SD** |  |  | 205 ± 88 | 5 ± 2 | 131 ± 6 | | 99 ± 7 | 140 ± 55 | 89 ± 7 | 16 ± 12 | 98 ± 4 |
