# Supplementary material for: Haul-Out Behaviour of the World's Northernmost Population of Harbour Seals (Phoca vitulina) throughout the Year
Source: PLoS One. 2014 Jan 22;9(1):e86055. doi: 10.1371/journal.pone.0086055 (PMC3899210; doi:10.1371/journal.pone.0086055)
Supplement: Table S3 — AICc table for time since previous haul-out event. The corrected Akaike information criterion (AICc), change in AICc and weight of the AICc for the different GAMM models for the time since previous haul-out event for the 60 harbour seals equipped with Satellite-Relay Data Loggers (SRDLs) in Svalbard, Norway in 2009 and 2010. Ytag is the year of tagging and maturity indicates whether the seal was a pup, immature or mature. (DOCX) [file pone.0086055.s006.docx]

| **Model structure** | **AICc** | **ΔAICc** | **AICc_w_** |
| --- | --- | --- | --- |
| *f*(month)+maturity+(1\|id) | 29165.49 | 0.00 | 0.47 |
| *f*(month)+(1\|id) | 29165.59 | 0.10 | 0.45 |
| *f*(month)+maturity+ytag+(1\|id) | 29169.21 | 3.72 | 0.07 |
| *f*(month)+maturity+sex+ytag+(1\|id) | 29173.01 | 7.52 | 0.01 |
| *f*(month, by=ytag)+maturity+sex+(1\|id) | 29176.60 | 11.11 | 0.00 |
| *f*(month, by=sex)+maturity+ytag+(1\|id) | 29177.66 | 12.17 | 0.00 |
| *f*(month, by=maturity)+sex+ytag+(1\|id) | 29189.39 | 23.90 | 0.00 |
