# Supplementary material for: Haul-Out Behaviour of the World's Northernmost Population of Harbour Seals (Phoca vitulina) throughout the Year
Source: PLoS One. 2014 Jan 22;9(1):e86055. doi: 10.1371/journal.pone.0086055 (PMC3899210; doi:10.1371/journal.pone.0086055)
Supplement: Table S4 — AICc table for use of off-shore ice as a haul-out platform. The corrected Akaike information criterion (AICc), change in AICc and weight of the AICc for the different GAMM models for the use of off-shore ice as a haul-out platform by the 60 harbour seals equipped with Satellite-Relay Data Loggers (SRLDs) in Svalbard, Norway in 2009 and 2010. Ytag is the year of tagging and maturity indicates whether the seal was a pup, immature or mature. (DOCX) [file pone.0086055.s007.docx]

| **Model structure** | **AICc** | **ΔAICc** | **AICc_w_** |
| --- | --- | --- | --- |
| *f*(month, by=ytag)+maturity+(1\|id) | 451.44 | 0.00 | 0.61 |
| *f*(month, by=ytag)+maturity+sex+(1\|id) | 453.09 | 1.65 | 0.27 |
| *f*(month, by=ytag)+(1\|id) | 454.56 | 3.12 | 0.13 |
| *f*(month, by=maturity)+ytag+(1\|id) | 571.11 | 119.67 | 0.00 |
| *f*(month)+maturity+ytag+(1\|id) | 571.42 | 119.98 | 0.00 |
| *f*(month, by=age)+ytag+maturity+(1\|id) | 577.49 | 126.05 | 0.00 |
