# Supplementary material for: Haul-Out Behaviour of the World's Northernmost Population of Harbour Seals (Phoca vitulina) throughout the Year
Source: PLoS One. 2014 Jan 22;9(1):e86055. doi: 10.1371/journal.pone.0086055 (PMC3899210; doi:10.1371/journal.pone.0086055)
Supplement: Table S5 — Percentage of haul-out events on off-shore ice. Percentage of haul-out events each month that took place on off-shore ice (mean ± bootstrapped 95% CI) for each maturity group by year for the 60 harbour seals equipped with Satellite-Relay Data Loggers (SRDLs) in Svalbard, Norway in 2009 and 2010. (DOCX) [file pone.0086055.s008.docx]

|  | **Pup** | | **Immature** | | **Mature** | |
| --- | --- | --- | --- | --- | --- | --- |
|  | **2009** | **2010** | **2009** | **2010** | **2009** | **2010** |
| **Sept** | 0 ± 0 | 0 ± 0 | 0 ± 0 | 0 ± 0 | 0 ± 0 | 0 ± 0 |
| **Oct** | 0 ± 0 | 0 ± 0 | 0 ± 0 | 0 ± 0 | 0 ± 0 | 0 ± 0 |
| **Nov** | 0 ± 1 | 18 ± 18 | 1 ± 2 | 0 ± 1 | 0 ± 0 | 0 ± 0 |
| **Dec** | 4 ± 5 | 79 ± 32 | 2 ± 3 | 10 ± 15 | 6 ± 5 | 12 ± 10 |
| **Jan** | 15 ± 17 | 99 ± 2 | 13 ± 12 | 37 ± 29 | 4 ± 4 | 50 ± 41 |
| **Feb** | 46 ± 29 | 100 ± 0 | 48 ± 24 | 91 ± 9 | 5 ± 6 | 88 ± 17 |
| **Mar** | 57 ± 34 | 93 ± 13 | 67 ± 53 | 68 ± 18 | 46 ± 21 | 46 ± 13 |
| **Apr** | 66 ± 34 | 89 ± 21 | 39 ± 29 | 35 ± 43 | 34 ± 22 | 29 ± 16 |
| **May** | 11 ± 14 | 0 ± 0 | 0 ± 0 | 0 ± 0 | 0 ± 0 | 0 ± 0 |
| **Jun** | 0 ± 0 | 0 ± 0 | 0 ± 0 | 0 ± 0 | 0 ± 0 | 0 ± 0 |
