# Supplementary material for: Haul-Out Behaviour of the World's Northernmost Population of Harbour Seals (Phoca vitulina) throughout the Year
Source: PLoS One. 2014 Jan 22;9(1):e86055. doi: 10.1371/journal.pone.0086055 (PMC3899210; doi:10.1371/journal.pone.0086055)
Supplement: Table S6 — Haul-out probability. GAMM model results for haul-out probability for the 60 harbour seals equipped with Satellite-Relay Data Loggers (SRDLs) in Svalbard, Norway in 2009 and 2010, showing the estimated degrees of freedom (edf) and p-values for the smooth terms, the variance of the random effect and the value of temporal autocorrelation (phi). The estimate (est), 95% CI and p-values are provided for the linear terms. Values of 0.5 indicate a 50/50 chance of hauling out vs not hauling out, with values beneath 0.5 indicating a greater probability of not hauling out and values over 0.5 indicating a greater probably of hauling out. The reference levels are: maturity = immature seals; sex = female; year of tagging (ytag) = 2009 (the first year of tagging) and; light = total darkness (sun <12 degrees below horizon), except where stated otherwise. (DOCX) [file pone.0086055.s009.docx]

|  |  | **Sept** | **Oct** | **Nov** | **Dec** | **Jan** | **Feb** | **Mar** | **Apr** | **May** | **Jun** |
| --- | --- | --- | --- | --- | --- | --- | --- | --- | --- | --- | --- |
| **Temperature - pups** | **edf** | 4.6670 | 1.0000 | 2.1390 | 1.0050 | 3.3720 | 1.7950 | 3.0600 | 1.5290 | 1.6950 | 1.0000 |
|  | **p-value** | 0.1185 | 0.3523 | <0.0001 | 0.1255 | 0.0362 | 0.6202 | 0.0047 | 0.0350 | 0.2851 | 0.2045 |
| **Temperature - immatures** | **edf** | 3.2560 | 1.6840 | 3.5260 | 3.3390 | 4.2630 | 1.0000 | 1.0000 | 2.3650 | 1.0000 | 1.0000 |
|  | **p-value** | 0.0827 | 0.0003 | <0.0001 | 0.0027 | 0.0929 | 0.0822 | 0.5669 | 0.0899 | 0.1142 | 0.5424 |
| **Temperature - matures** | **edf** | 3.0970 | 2.3220 | 5.2710 | 3.4510 | 1.0000 | 1.0000 | 1.0000 | 1.0000 | 1.0000 | 1.0000 |
|  | **p-value** | 0.0090 | 0.2330 | <0.0001 | 0.0042 | 0.0012 | 0.3519 | 0.4389 | <0.0001 | 0.6506 | 0.8487 |
| **Fraction of the moon illuminated** | **edf** | 1.0030 | 5.9600 | 4.9400 | 6.3850 | 1.0000 | 1.0000 | 2.6570 | 4.9760 | 3.5110 | 3.9270 |
|  | **p-value** | 0.3719 | 0.0007 | <0.0001 | <0.0001 | 0.0038 | 0.7339 | <0.0001 | 0.0002 | 0.0016 | 0.0004 |
| **Solar hour** | **edf** | 5.7540 | 5.4790 | 2.6190 | 1.1800 | 2.3110 | 1.6090 | 2.7000 | 3.7140 | 2.2900 | 2.3480 |
|  | **p-value** | <0.0001 | <0.0001 | 0.0018 | 0.1675 | 0.0375 | 0.0563 | <0.0001 | <0.0001 | 0.0002 | 0.0186 |
| **Time to low tide** | **edf** | 6.7950 | 6.4240 | 6.4870 | 6.5390 | 5.3040 | 4.6260 | 3.4910 | 5.7450 | 3.9000 | 6.2410 |
|  | **p-value** | <0.0001 | <0.0001 | <0.0001 | <0.0001 | <0.0001 | 0.0020 | 0.0143 | <0.0001 | <0.0001 | <0.0001 |
| **Air pressure at sea level** | **edf** | 4.0220 | 3.0540 | 1.0030 | 4.9680 | 2.0960 | 1.0000 | 1.0000 | 1.0000 | 1.0000 | 2.7030 |
|  | **p-value** | <0.0001 | 0.0003 | 0.8310 | <0.0001 | 0.0860 | 0.0004 | <0.0001 | 0.0005 | 0.0613 | <0.0001 |
| **Air pressure Δ 12 hrs** | **edf/est** | 3.3420 | 3.0130 | 1.0000 | 0.4793 | 1.0000 | 3.7690 | 0.4979 | 2.3070 | 0.4609 | 1.5150 |
|  | **95% CI** |  |  |  | ± 0.0224 |  |  | ± 0.0395 |  | ± 0.0553 |  |
|  | **p-value** | 0.0171 | 0.0023 | 0.2661 | 0.0712 | 0.0004 | 0.0043 | 0.9181 | 0.0924 | 0.1733 | 0.4697 |
| **Air pressure Δ 24 hrs** | **edf/est** | 4.5690 | 1.0000 | 2.6870 | 4.7730 | 1.0000 | 1.0000 | 0.4927 | 1.0000 | 1.0000 | 1.9810 |
|  | **95% CI** |  |  |  |  |  |  | ± 0.0431 |  |  |  |
|  | **p-value** | 0.0018 | 0.0556 | 0.4884 | <0.0001 | 0.8776 | 0.2079 | 0.7416 | 0.4005 | 0.3163 | 0.2725 |
| **Air pressure Δ 3 hrs** | **est** | 0.4945 | 0.4458 | 0.4624 | 0.4874 | 0.4607 | 0.4628 | 0.5015 | 0.4567 | 0.4459 | 0.4966 |
|  | **95% CI** | ± 0.0183 | ± 0.0152 | ± 0.0207 | ± 0.0192 | ± 0.0222 | ± 0.0329 | ± 0.0257 | ± 0.0259 | ± 0.0456 | ± 0.0595 |
|  | **p-value** | 0.5537 | <0.0001 | 0.0004 | 0.2021 | 0.0006 | 0.0286 | 0.9114 | 0.0012 | 0.0228 | 0.9107 |
| **Latitude** | **est** | 0.5481 | 0.4089 | 0.5706 | 0.6189 | 0.5670 | 0.6724 | 0.4273 | 0.5720 | 0.5394 | 0.3614 |
|  | **95% CI** | ± 0.0806 | ± 0.0514 | ± 0.0551 | ± 0.0553 | ± 0.0386 | ± 0.054 | ± 0.0493 | ± 0.0572 | ± 0.0555 | ± 0.0965 |
|  | **p-value** | 0.2418 | 0.0009 | 0.0123 | <0.0001 | 0.0007 | <0.001 | 0.0051 | 0.0138 | 0.1627 | 0.0135 |
| **Longitude** | **est** | 0.5688 | 0.4015 | 0.6368 | 0.6453 | 0.6126 | 0.6845 | 0.5529 | 0.6583 | 0.6465 | 0.7825 |
|  | **95% CI** | ± 0.0484 | ± 0.0512 | ± 0.0544 | ± 0.0546 | ± 0.0381 | ± 0.0513 | ± 0.0451 | ± 0.0436 | ± 0.0485 | ± 0.0799 |
|  | **p-value** | 0.0055 | 0.0003 | <0.0001 | <0.0001 | <0.0001 | <0.0001 | 0.0216 | <0.0001 | <0.0001 | <0.0001 |
| **Sex (male)** | **est** | 0.4826 | 0.5481 | 0.5265 | 0.4755 | 0.5324 | 0.7436 | 0.6043 | 0.6457 | 0.6107 | 0.5055 |
|  | **95% CI** | ± 0.0639 | ± 0.0643 | ± 0.0755 | ± 0.0857 | ± 0.063 | ± 0.1393 | ± 0.0932 | ± 0.1784 | ± 0.0795 | ± 0.2911 |
|  | **p-value** | 0.5981 | 0.1415 | 0.4921 | 0.5832 | 0.3137 | 0.0011 | 0.0285 | 0.1077 | 0.0066 | 0.9738 |

|  |  | **Sept** | **Oct** | **Nov** | **Dec** | **Jan** | **Feb** | **Mar** | **Apr** | **May** | **Jun** |
| --- | --- | --- | --- | --- | --- | --- | --- | --- | --- | --- | --- |
| **Maturity (mature seals)** | **est** | 0.5096 | 0.4781 | 0.5187 | 0.5264 | 0.5453 | 0.6628 | 0.5810 | 0.6117 | 0.5956 | 0.4357 |
|  | **95% CI** | ± 0.0824 | ± 0.0787 | ± 0.0957 | ± 0.1062 | ± 0.0801 | ± 0.1623 | ± 0.0956 | ± 0.1813 | ± 0.0728 | ± 0.2848 |
|  | **p-value** | 0.8197 | 0.5924 | 0.7041 | 0.6281 | 0.2682 | 0.0494 | 0.0962 | 0.2253 | 0.0104 | 0.7301 |
| **Maturity (pups)** | **est** | 0.3709 | 0.4049 | 0.4456 | 0.5236 | 0.6297 | 0.7089 | 0.6257 | 0.5581 | 0.4855 | 0.6686 |
|  | **95% CI** | ± 0.0711 | ± 0.0761 | ± 0.0924 | ± 0.1125 | ± 0.0777 | ± 0.1589 | ± 0.092 | ± 0.191 | ± 0.0852 | ± 0.3516 |
|  | **p-value** | 0.0012 | 0.0216 | 0.2684 | 0.6837 | 0.0013 | 0.0114 | 0.0079 | 0.5565 | 0.7420 | 0.3492 |
| **Ytag (second year)** | **est** | 0.4396 | 0.4692 | 0.3029 | 0.4547 | 0.5689 | 0.4484 | 0.6627 | 0.5847 | 0.6735 | 0.5462 |
|  | **95% CI** | ± 0.0752 | ± 0.0659 | ± 0.0660 | ± 0.0907 | **±** 0.0715 | ± 0.1348 | ± 0.0911 | ± 0.1650 | ± 0.0739 | ± 0.2342 |
|  | **p-value** | 0.1289 | 0.3679 | <0.0001 | 0.3442 | 0.0591 | 0.4814 | 0.0006 | 0.3141 | <0.0001 | 0.7096 |
| **Light** | **est** | 0.5580 | 0.5795 | NA | NA | NA | 0.4209 | 0.4777 | ref | NA | NA |
|  | **95% CI** | ± 0.0445 | ± 0.0283 | NA | NA | NA | ± 0.0549 | ± 0.0492 | ref | NA | NA |
|  | **p-value** | 0.0108 | <0.0001 | NA | NA | NA | 0.0066 | 0.3780 | ref | NA | NA |
| **Nautical dawn** | **est** | 0.4924 | 0.5224 | 0.5180 | 0.5044 | 0.4771 | 0.4348 | 0.4910 | 0.4889 | NA | NA |
|  | **95% CI** | ± 0.0441 | ± 0.0264 | ± 0.0222 | ± 0.0244 | ± 0.0331 | ± 0.0365 | ± 0.0441 | ± 0.0483 | NA | NA |
|  | **p-value** | 0.7387 | 0.0962 | 0.1124 | 0.7255 | 0.1777 | 0.0006 | 0.6918 | 0.6548 | NA | NA |
| **Nautical dusk** | **est** | 0.5127 | 0.5200 | 0.4986 | 0.5141 | 0.4944 | 0.4638 | 0.5021 | 0.5139 | NA | NA |
|  | **95% CI** | ± 0.0425 | ± 0.0245 | ± 0.0238 | ± 0.0248 | ± 0.0341 | ± 0.0341 | ± 0.0417 | ± 0.0442 | NA | NA |
|  | **p-value** | 0.5589 | 0.1091 | 0.9063 | 0.2658 | 0.7490 | 0.0400 | 0.9227 | 0.5390 | NA | NA |
| **Light*latitude** | **est** | 0.4871 | 0.4886 | NA | NA | NA | 0.4859 | 0.5250 | ref | NA | NA |
|  | **95% CI** | ± 0.0623 | ± 0.0333 | NA | NA | NA | ± 0.0423 | ± 0.0307 | ref | NA | NA |
|  | **p-value** | 0.6871 | 0.5031 | NA | NA | NA | 0.5177 | 0.1104 | ref | NA | NA |
| **Nautical dawn*latitude** | **est** | 0.4941 | 0.5150 | 0.4985 | 0.4781 | 0.5168 | 0.5135 | 0.5079 | 0.5421 | NA | NA |
|  | **95% CI** | ± 0.0635 | ± 0.0315 | ± 0.0231 | ± 0.0231 | ± 0.0198 | ± 0.0254 | ± 0.0285 | ± 0.0373 | NA | NA |
|  | **p-value** | 0.8554 | 0.3509 | 0.9024 | 0.0648 | 0.0968 | 0.2970 | 0.5867 | 0.0271 | NA | NA |
| **Nautical dusk*latitude** | **est** | 0.4870 | 0.4965 | 0.5159 | 0.4811 | 0.5070 | 0.4902 | 0.5240 | 0.5230 | NA | NA |
|  | **95% CI** | ± 0.0604 | ± 0.0299 | ± 0.0255 | ± 0.0237 | ± 0.0191 | ± 0.0214 | ± 0.0274 | ± 0.0324 | NA | NA |
|  | **p-value** | 0.6760 | 0.8193 | 0.2226 | 0.1182 | 0.4797 | 0.3735 | 0.0862 | 0.1640 | NA | NA |
| **Variance of random effect (id)** |  | 0.1729 | 0.1470 | 0.1855 | 0.2255 | 0.0000 | 0.3939 | 0.0000 | 0.4086 | 0.0000 | 1.4358 |
| **Phi** |  | 0.8063 | 0.7916 | 0.8110 | 0.8132 | 0.8203 | 0.8051 | 0.8284 | 0.8179 | 0.8023 | 0.8381 |
| **Normalized residuals vs fitted values** | **F-value** | 0.07 | 0.02 | 2.16 | 3.83 | 0.03 | 0.79 | 0.99 | 1.69 | 1.20 | 0.30 |
|  | **p-value** | 0.8 | 0.9 | 0.1 | 0.1 | 0.9 | 0.4 | 0.3 | 0.2 | 0.3 | 0.6 |
