# Supplementary material for: Haul-Out Behaviour of the World's Northernmost Population of Harbour Seals (Phoca vitulina) throughout the Year
Source: PLoS One. 2014 Jan 22;9(1):e86055. doi: 10.1371/journal.pone.0086055 (PMC3899210; doi:10.1371/journal.pone.0086055)
Supplement: Table S7 — Haul-out duration. Cox Proportional Hazard model results for haul-out duration for the 60 harbour seals equipped with Satellite-Relay Data Loggers (SRDLs) in Svalbard, Norway in 2009 and 2010, showing the hazard ratio (exp(coef)), 95% CI and the p-values for each covariate. For the smooth terms only the p-values are given. The reference levels are: maturity = immature seals; sex = female; year of tagging (ytag) = 2009 (first year of tagging); light = total darkness (sun <12 degrees below horizon), except where stated otherwise and; haul-out substrate = land, except where stated otherwise. (DOCX) [file pone.0086055.s010.docx]

|  |  | **Sept** | **Oct** | **Nov** | **Dec** | **Jan** | **Feb** | **Mar** | **Apr** | **May** | **Jun** |
| --- | --- | --- | --- | --- | --- | --- | --- | --- | --- | --- | --- |
| **SMOOTH TERMS** |  |  |  |  |  |  |  |  |  |  |  |
| **Time to previous haul-out event** | **p-value** | <0.0001 | <0.0001 | 0.0730 | 0.1500 | 0.0500 | 0.0110 | 0.1400 | 0.1200 | 0.2700 | 0.6600 |
| **Solar hour** | **p-value** | <0.0001 | <0.0001 | 0.4600 | 0.1800 | 0.3500 | 0.1500 | 0.0910 | 0.0480 | 0.0020 | 0.0280 |
| **Time to low tide** | **p-value** | <0.0001 | <0.0001 | <0.0001 | 0.0002 | 0.4100 | 0.3400 | 0.6000 | 0.8800 | <0.0001 | <0.0001 |
| **LINEAR TERMS** |  |  |  |  |  |  |  |  |  |  |  |
| **Temperature** | **exp (coef)** | 1.1200 | 0.9500 | 1.0530 | 1.0640 | 1.0560 | 1.1590 | 1.2030 | 1.3420 | 1.0890 | 1.5200 |
|  | **lower 95% CI** | 0.9260 | 0.8210 | 0.9080 | 0.9400 | 0.9430 | 0.9573 | 0.9705 | 1.0839 | 0.8142 | 0.9671 |
|  | **upper 95% CI** | 1.3540 | 1.0990 | 1.2220 | 1.2050 | 1.1840 | 1.4020 | 1.4900 | 1.6610 | 1.4560 | 2.3880 |
|  | **p-value** | 0.2400 | 0.4900 | 0.4900 | 0.3200 | 0.3500 | 0.1300 | 0.0920 | 0.0069 | 0.5700 | 0.0700 |
| **Air pressure** | **exp (coef)** | 0.8840 | 0.9150 | 0.9440 | 1.0020 | 1.0290 | 1.0030 | 0.8750 | 0.9250 | 1.0800 | 0.7040 |
|  | **lower 95% CI** | 0.8136 | 0.8480 | 0.8850 | 0.9290 | 0.9190 | 0.8497 | 0.7421 | 0.8115 | 0.9329 | 0.5610 |
|  | **upper 95% CI** | 0.9610 | 0.9870 | 1.0070 | 1.0810 | 1.1530 | 1.1840 | 1.0320 | 1.0550 | 1.2510 | 0.8840 |
|  | **p-value** | 0.0038 | 0.0210 | 0.0820 | 0.9600 | 0.6200 | 0.9700 | 0.1100 | 0.2400 | 0.3000 | 0.0025 |
| **Air pressure Δ 3 hrs** | **exp (coef)** | 1.1260 | 1.3030 | 1.0420 | 1.0270 | 1.1550 | 0.7970 | 0.6820 | 1.0890 | 0.6270 | 0.5350 |
|  | **lower 95% CI** | 0.9044 | 1.0660 | 0.7840 | 0.7440 | 0.8800 | 0.4912 | 0.4662 | 0.6874 | 0.3529 | 0.2564 |
|  | **upper 95% CI** | 1.4010 | 1.5930 | 1.3860 | 1.4180 | 1.5170 | 1.2940 | 0.9970 | 1.7240 | 1.1140 | 1.1180 |
|  | **p-value** | 0.2900 | 0.0097 | 0.7700 | 0.8700 | 0.3000 | 0.3600 | 0.0480 | 0.7200 | 0.1100 | 0.0960 |
| **Air pressure Δ 12 hrs** | **exp (coef)** | 0.9780 | 0.8910 | 0.9100 | 0.9760 | 1.3610 | 1.4020 | 1.6880 | 0.8580 | 1.8560 | 1.6090 |
|  | **lower 95% CI** | 0.7379 | 0.7090 | 0.6360 | 0.6480 | 0.9520 | 0.7258 | 1.0348 | 0.4573 | 0.9763 | 0.6508 |
|  | **upper 95% CI** | 1.2970 | 1.1200 | 1.3000 | 1.4700 | 1.9460 | 2.7070 | 2.7540 | 1.6100 | 3.5270 | 3.9800 |
|  | **p-value** | 0.8800 | 0.3200 | 0.6000 | 0.9100 | 0.0910 | 0.3100 | 0.0360 | 0.6300 | 0.0590 | 0.3000 |
| **Air pressure Δ 24 hrs** | **exp (coef)** | 1.0550 | 1.0860 | 1.1210 | 1.1470 | 1.0520 | 0.8500 | 0.9340 | 1.2750 | 0.8050 | 1.1460 |
|  | **lower 95% CI** | 0.8720 | 0.9240 | 0.8890 | 0.9200 | 0.7920 | 0.5510 | 0.6221 | 0.8696 | 0.5049 | 0.6553 |
|  | **upper 95% CI** | 1.2760 | 1.2770 | 1.4130 | 1.4300 | 1.3980 | 1.3130 | 1.4040 | 1.8690 | 1.2820 | 2.0030 |
|  | **p-value** | 0.5800 | 0.3200 | 0.3300 | 0.2200 | 0.7300 | 0.4600 | 0.7400 | 0.2100 | 0.3600 | 0.6300 |
| **Fraction of the moon illuminated** | **exp (coef)** | 1.0150 | 1.0050 | 0.8670 | 0.9550 | 0.9510 | 1.0650 | 1.1190 | 1.0980 | 1.0680 | 1.1430 |
|  | **lower 95% CI** | 0.9589 | 0.9380 | 0.7990 | 0.8860 | 0.8670 | 0.9281 | 0.9891 | 0.9740 | 0.9219 | 0.9645 |
|  | **upper 95% CI** | 1.0750 | 1.0760 | 0.9400 | 1.0280 | 1.0450 | 1.2200 | 1.2670 | 1.2370 | 1.2380 | 1.3550 |
|  | **p-value** | 0.6000 | 0.8900 | 0.0006 | 0.2200 | 0.3000 | 0.3700 | 0.0740 | 0.1300 | 0.3800 | 0.1200 |

|  |  | **Sept** | **Oct** | **Nov** | **Dec** | **Jan** | **Feb** | **Mar** | **Apr** | **May** | **Jun** |
| --- | --- | --- | --- | --- | --- | --- | --- | --- | --- | --- | --- |
| **Latitude** | **exp (coef)** | 1.3920 | 1.1960 | 1.0620 | 0.9350 | 1.0230 | 0.8510 | 0.9620 | 0.8530 | 1.4990 | 2.1480 |
|  | **lower 95% CI** | 0.7402 | 1.0050 | 0.8890 | 0.7910 | 0.8650 | 0.6725 | 0.7444 | 0.6998 | 1.1641 | 1.5967 |
|  | **upper 95% CI** | 2.6190 | 1.4230 | 1.2690 | 1.1060 | 1.2000 | 1.0770 | 1.2420 | 1.0400 | 1.9300 | 2.8910 |
|  | **p-value** | 0.3000 | 0.0440 | 0.5100 | 0.4300 | 0.7900 | 0.1800 | 0.7600 | 0.1200 | 0.0017 | <0.0001 |
| **Longitude** | **exp (coef)** | 1.2100 | 1.2750 | 0.9930 | 1.0470 | 0.9930 | 0.8250 | 0.8960 | 0.9480 | 1.1720 | 1.7390 |
|  | **lower 95% CI** | 1.0443 | 1.0650 | 0.8050 | 0.8760 | 0.8420 | 0.6384 | 0.7272 | 0.8079 | 0.9022 | 1.2588 |
|  | **upper 95% CI** | 1.4020 | 1.5260 | 1.2260 | 1.2510 | 1.1700 | 1.0650 | 1.1040 | 1.1110 | 1.5230 | 2.4030 |
|  | **p-value** | 0.0110 | 0.0083 | 0.9500 | 0.6100 | 0.9300 | 0.1400 | 0.3000 | 0.5100 | 0.2300 | 0.0008 |
| **Sex (males)** | **exp (coef)** | 1.1500 | 0.9230 | 1.0560 | 0.9500 | 1.0190 | 0.6220 | 0.8820 | 0.9380 | 0.8460 | 0.8880 |
|  | **lower 95% CI** | 0.9971 | 0.7910 | 0.8890 | 0.7850 | 0.7950 | 0.4311 | 0.6056 | 0.6530 | 0.5799 | 0.5767 |
|  | **upper 95% CI** | 1.3270 | 1.0770 | 1.2540 | 1.1500 | 1.3070 | 0.8970 | 1.2850 | 1.3470 | 1.2340 | 1.3680 |
|  | **p-value** | 0.0550 | 0.3000 | 0.5400 | 0.6000 | 0.8800 | 0.0110 | 0.5100 | 0.7300 | 0.3900 | 0.5900 |
| **Maturity (mature seals)** | **exp (coef)** | 0.9860 | 0.9730 | 1.1430 | 0.9420 | 0.9000 | 0.8540 | 1.0140 | 0.7960 | 0.6550 | 0.5640 |
|  | **lower 95% CI** | 0.8223 | 0.8010 | 0.9150 | 0.7420 | 0.6750 | 0.5988 | 0.6880 | 0.5534 | 0.4697 | 0.3433 |
|  | **upper 95% CI** | 1.1810 | 1.1820 | 1.4270 | 1.1940 | 1.1990 | 1.2170 | 1.4940 | 1.1440 | 0.9140 | 0.9260 |
|  | **p-value** | 0.8800 | 0.7800 | 0.2400 | 0.6200 | 0.4700 | 0.3800 | 0.9400 | 0.2200 | 0.0130 | 0.0240 |
| **Maturity (pups)** | **exp (coef)** | 1.1380 | 1.0510 | 0.8240 | 0.8360 | 0.6580 | 0.5220 | 0.8210 | 0.7850 | 0.7190 | 0.9050 |
|  | **lower 95% CI** | 0.9478 | 0.8510 | 0.6480 | 0.6470 | 0.4790 | 0.3590 | 0.5524 | 0.5319 | 0.4873 | 0.5510 |
|  | **upper 95% CI** | 1.3660 | 1.2970 | 1.0460 | 1.0800 | 0.9050 | 0.7600 | 1.2190 | 1.1600 | 1.0600 | 1.4860 |
|  | **p-value** | 0.1700 | 0.6500 | 0.1100 | 0.1700 | 0.0099 | 0.0007 | 0.3300 | 0.2200 | 0.0960 | 0.6900 |
| **Year** | **exp (coef)** | 0.9830 | 1.0440 | 0.9690 | 0.7800 | 0.8910 | 1.1000 | 0.6990 | 0.5860 | 0.7410 | 1.7040 |
|  | **lower 95% CI** | 0.8013 | 0.8860 | 0.7770 | 0.6270 | 0.6740 | 0.7285 | 0.4700 | 0.4045 | 0.5123 | 1.0048 |
|  | **upper 95% CI** | 1.2050 | 1.2290 | 1.2090 | 0.9710 | 1.1800 | 1.6620 | 1.0390 | 0.8490 | 1.0710 | 2.8900 |
|  | **p-value** | 0.8700 | 0.6100 | 0.7800 | 0.0260 | 0.4200 | 0.6500 | 0.0770 | 0.0048 | 0.1100 | 0.0480 |
| **Light** | **exp (coef)** | 0.7990 | 0.7130 | NA | NA | NA | 1.8220 | 1.3520 | ref | NA | NA |
|  | **lower 95% CI** | 0.4035 | 0.4990 | NA | NA | NA | 0.8851 | 0.7778 | ref | NA | NA |
|  | **upper 95% CI** | 1.5810 | 1.0180 | NA | NA | NA | 3.7500 | 2.3510 | ref | NA | NA |
|  | **p-value** | 0.5200 | 0.0620 | NA | NA | NA | 0.1000 | 0.2800 | ref | NA | NA |
| **Nautical dawn** | **exp (coef)** | 0.4570 | 0.9610 | 0.9100 | 1.0850 | 1.0700 | 1.6540 | 1.6320 | 0.7880 | NA | NA |
|  | **lower 95% CI** | 0.2081 | 0.6860 | 0.6740 | 0.7250 | 0.6680 | 0.9281 | 0.1012 | 0.3205 | NA | NA |
|  | **upper 95% CI** | 1.0030 | 1.3460 | 1.2270 | 1.6240 | 1.7140 | 2.9490 | 2.6330 | 1.9370 | NA | NA |
|  | **p-value** | 0.0510 | 0.8200 | 0.5400 | 0.6900 | 0.7800 | 0.0880 | 0.0450 | 0.6000 | NA | NA |

|  |  | **Sept** | **Oct** | **Nov** | **Dec** | **Jan** | **Feb** | **Mar** | **Apr** | **May** | **Jun** |
| --- | --- | --- | --- | --- | --- | --- | --- | --- | --- | --- | --- |
| **Nautical dusk** | **exp (coef)** | 0.7430 | 1.3150 | 0.8420 | 0.9330 | 1.1630 | 1.0820 | 1.3960 | 1.3320 | NA | NA |
|  | **lower 95% CI** | 0.4074 | 0.9740 | 0.6170 | 0.6630 | 0.7360 | 0.6439 | 0.8983 | 0.7292 | NA | NA |
|  | **upper 95% CI** | 1.3550 | 1.7740 | 1.1500 | 1.3130 | 1.8400 | 1.8180 | 2.1700 | 2.4330 | NA | NA |
|  | **p-value** | 0.3300 | 0.0730 | 0.2800 | 0.6900 | 0.5200 | 0.7700 | 0.1400 | 0.3500 | NA | NA |
| **Light*latitude** | **exp (coef)** | 0.7450 | 1.0450 | NA | NA | NA | 0.9740 | 1.0120 | ref | NA | NA |
|  | **lower 95% CI** | 0.3994 | 0.8700 | NA | NA | NA | 0.5776 | 0.8337 | ref | NA | NA |
|  | **upper 95% CI** | 1.3910 | 1.2550 | NA | NA | NA | 1.6430 | 1.2280 | ref | NA | NA |
|  | **p-value** | 0.3600 | 0.6400 | NA | NA | NA | 0.9200 | 0.9100 | ref | NA | NA |
| **Nautical dawn*latitude** | **exp (coef)** | 0.6070 | 0.9540 | 0.9010 | 1.0660 | 0.7570 | 1.0550 | 0.9010 | 2.6400 | NA | NA |
|  | **lower 95% CI** | 0.2927 | 0.7760 | 0.7340 | 0.7800 | 0.6060 | 0.6852 | 0.7462 | 1.2834 | NA | NA |
|  | **upper 95% CI** | 1.2600 | 1.1720 | 1.1070 | 1.4580 | 0.9460 | 1.6250 | 1.0870 | 5.4290 | NA | NA |
|  | **p-value** | 0.1800 | 0.6500 | 0.3200 | 0.6900 | 0.0140 | 0.8100 | 0.2800 | 0.0083 | NA | NA |
| **Nautical dusk*latitude** | **exp (coef)** | 0.8860 | 1.0230 | 0.7920 | 1.1010 | 0.7640 | 1.0310 | 1.0660 | 0.7710 | NA | NA |
|  | **lower 95% CI** | 0.4691 | 0.8640 | 0.6280 | 0.8530 | 0.6540 | 0.8466 | 0.7504 | 0.4966 | NA | NA |
|  | **upper 95% CI** | 1.6740 | 1.2110 | 0.9990 | 1.4220 | 0.8930 | 1.2560 | 1.5150 | 1.1980 | NA | NA |
|  | **p-value** | 0.7100 | 0.7900 | 0.0490 | 0.4600 | 0.0007 | 0.7600 | 0.7200 | 0.2500 | NA | NA |
| **Haul-out substrate (shore-fast ice)** | **exp (coef)** | NA | NA | 0.8030 | ref | ref | ref | ref | ref | 1.2180 | 1.1130 |
|  | **lower 95% CI** | NA | NA | 0.6880 | ref | ref | ref | ref | ref | 0.8117 | 0.5898 |
|  | **upper 95% CI** | NA | NA | 0.9390 | ref | ref | ref | ref | ref | 1.8280 | 2.0990 |
|  | **p-value** | NA | NA | 0.0058 | ref | ref | ref | ref | ref | 0.3400 | 0.7400 |
| **Haul-out substrate (off-shore ice)** | **exp (coef)** | NA | NA | 0.7340 | 0.9200 | 0.9380 | 0.8750 | 0.8880 | 1.2570 | 0.3570 | NA |
|  | **lower 95% CI** | NA | NA | 0.4670 | 0.6790 | 0.6750 | 0.6164 | 0.6520 | 0.9422 | 0.1526 | NA |
|  | **upper 95% CI** | NA | NA | 1.1510 | 1.2470 | 1.3050 | 1.2410 | 1.2100 | 1.6770 | 0.8330 | NA |
|  | **p-value** | NA | NA | 0.1800 | 0.5900 | 0.7100 | 0.4500 | 0.4500 | 0.1200 | 0.0170 | NA |
| **Frailty (id)** | **df** | 0.3800 | 3.4000 | 2.4900 | 2.0100 | -0.1300 | -2.1700 | -2.6500 | -2.2100 | -1.8100 | -2.4500 |
|  | **p-value** | <0.0001 | <0.0001 | <0.0001 | <0.0001 | 0.0007 | 0.0038 | 0.0170 | 0.0078 | <0.0001 | 0.0370 |
